# Noma Affected Children from Niger Have Distinct Oral Microbial Communities Based on High-Throughput Sequencing of 16S rRNA Gene Fragments

**DOI:** 10.1371/journal.pntd.0003240

**Published:** 2014-12-04

**Authors:** Katrine L. Whiteson, Vladimir Lazarevic, Manuela Tangomo-Bento, Myriam Girard, Heather Maughan, Didier Pittet, Patrice Francois, Jacques Schrenzel, the GESNOMA study group

**Affiliations:** 1 Genomic Research Laboratory, Department of Internal Medicine, Service of Infectious Diseases, University of Geneva Hospitals, Geneva, Switzerland; 2 Department of Molecular Biology and Biochemistry, University of California, Irvine, Irvine, California, United States of America; 3 Ronin Institute, Montclair, New Jersey, United States of America; 4 Infection Control Program and WHO Collaborating Centre on Patient Safety, University of Geneva Hospitals, Geneva, Switzerland; 5 Clinical Microbiology Laboratory, University of Geneva Hospitals, Geneva, Switzerland; University of Tennessee, United States of America

## Abstract

We aim to understand the microbial ecology of noma (cancrum oris), a devastating ancient illness which causes severe facial disfigurement in>140,000 malnourished children every year. The cause of noma is still elusive. A chaotic mix of microbial infection, oral hygiene and weakened immune system likely contribute to the development of oral lesions. These lesions are a plausible entry point for unidentified microorganisms that trigger gangrenous facial infections. To catalog bacteria present in noma lesions and identify candidate noma-triggering organisms, we performed a cross-sectional sequencing study of 16S rRNA gene amplicons from sixty samples of gingival fluid from twelve healthy children, twelve children suffering from noma (lesion and healthy sites), and twelve children suffering from Acute Necrotizing Gingivitis (ANG) (lesion and healthy sites). Relative to healthy individuals, samples taken from lesions in diseased mouths were enriched with *Spirochaetes* and depleted for *Proteobacteria*. Samples taken from healthy sites of diseased mouths had proportions of *Spirochaetes* and *Proteobacteria* that were similar to healthy control individuals. Samples from noma mouths did not have a higher abundance of *Fusobacterium*, casting doubt on its role as a causative agent of noma. Microbial communities sampled from noma and ANG lesions were dominated by the same *Prevotella intermedia* OTU, which was much less abundant in healthy sites sampled from the same mouths. Multivariate analysis confirmed that bacterial communities in healthy and lesion sites were significantly different. Several OTUs in the Orders Erysipelotrichales, Clostridiales, Bacteroidales, and Spirochaetales were identified as indicators of noma, suggesting that one or more microbes within these Orders is associated with the development of noma lesions. Future studies should include longitudinal sampling of viral and microbial components of this community, before and early in noma lesion development.

## Author Summary

Noma is a traumatic disease characterized by oral-facial lesions that often lead to severe disfigurement and ultimately shame and isolation from the community. Because the causes of noma are likely to be numerous, and reaching those who suffer from this illness is challenging, the etiology of noma remains ill-defined. Although it is known that oral hygiene and nutrition influence the development of noma, evidence suggests that one or more microbes play a crucial role in development of noma lesions. Previous studies have examined the DNA of microbes in lesions to determine which species are present and how their abundances differ between healthy mouth sites and noma lesions. These studies used techniques that were state-of-the-art at the time, though we know they likely only scratched the surface of the resident microbial diversity. Here we extend these studies by digging deeper to characterize a larger diversity of microbial species in noma and control samples, with the goal of better identifying which microbes are uniquely present or have altered abundances in noma lesions.

## Introduction

Noma, or *cancrum oris*, has caused devastating gangrenous oral-lesions throughout human history. It is a rapid and destructive infection that is thought to have a microbial origin in the context of poor immune function. Within 24–48 hours of the emergence of swelling in the cheek, often accompanied by gingivitis, gangrenous infection overtakes a region of the cheek, gums and facial bones. The ability of the infection to overcome structural barriers like muscle and bone is unusual. The term noma is derived from the Greek verb numein (to devour). It is a complex disease which arises most often in young children who are malnourished, exposed to infectious diseases, and lack access to clean water and dental hygiene. Noma has not been prevalent in developed countries since the 20^th^ century, with the exception of concentration camps in WWII, and isolated instances in Europe and the United States in people who are immunocompromised [1]. The WHO estimated a world-wide incidence of 140,000 cases per year in 1998, with 100,000 occurring in children who are 1–7 years old in sub-Saharan Africa [2]. The mortality rate is estimated to be 80–90%, though 770,000 people are thought to have survived the acute phase of infection. This may be an underestimate because noma occurs in remote parts of the world and is not often reported because of the great deal of shame associated with the disease. Current treatment includes hydration and nutrition, local disinfection of the wound, antibiotic therapy, and reconstructive surgery after the wounds have healed.

Noma is not considered an infectious disease. There have not been outbreaks reported in villages, and recurrence is rare. In one family, twins who both developed noma were affected in different years [3]. Attempts to infect mammals by inoculation of sub-gingival microbiota were only successful with the addition of steroid injections [4].

The exact etiology of noma is unknown. Several risk factors are thought to work together to increase the risk of developing the disease, including: 1) malnutrition 2) poor oral hygiene, potentially including oral lesions from gingivitis 3) a compromised immune system and 4) an unidentified triggering micro-organism, previously hypothesized to be a member of *Fusobacterium* or *Prevotella* [5]–[8]. The type of necrosis and the odor of the lesions are the main evidence supporting the long-held theory that a bacterium triggers the infection [1], [3], [6], [9].

Several limitations have hindered identification of the microbiological cause of noma. Because it develops rapidly in remote regions, obtaining reliable samples during early development of the disease is difficult. Earlier studies of noma relied on microbiological culturing of samples, which biases the results towards organisms that can grow in the culture conditions and may not reveal important members of the microbial community. Also, without knowing the composition of oral bacterial populations in healthy children, or children in similar circumstances but who do not have noma, it is difficult to separate disease-causing organisms from those that are common in the local population.

The recent revolution in DNA-based taxonomy methods enabled the discovery of the 99% of microorganisms that are difficult to grow in culture, but thrive in various environments, including the human body [1], [10]. Increased knowledge of these commensal microbial communities has refined our thinking about how microbes cause disease. For more than a century, medical doctors have focused on individual pathogens when studying infectious disease. In reality many infections may originate from, or be influenced by, the thriving communities of understudied commensal microbiota. In many cases, disease may arise from a disturbed microbial community, rather than a single pathogen [11], [12].

The obscure microbial origin of noma infection is well-suited for an investigation of how a shifted microbial community can result in disease. A single microbial trigger may not be revealed, but rather a combination of altered microbial abundances, which lead to disease in the context of malnutrition and a compromised immune system. The interplay with a background of malnutrition may mean that a commensal or opportunistic pathogen that is also present in healthy people triggers the disease. Ideally, microbial communities would be sampled before, during and after the appearance of noma wounds. This is still an elusive goal, as obtaining early samples is challenging under any circumstance, and becomes nearly impossible in rural areas where modern medicine is not accessible.

The GESNOMA group has led DNA-based efforts to identify the etiology of noma, having obtained and studied the bacterial communities in 413 samples over the last 10 years [9], [13], [14]. Sampled children in Niger included those deemed healthy, diagnosed with noma, and diagnosed with Acute Necrotizing Gingivitis (ANG), a related periodontal disease characterized by painful infections of the gums (also known as trench mouth) [15]. The ages of healthy controls were matched as closely as possible to those suffering from noma. Bacterial communities in these samples were characterized using DNA-based methods to learn about the epidemiology and microbiology of Noma [1], [9], [13], [14].

These recent GESNOMA studies suggest that the same (or very similar) bacteria are present in noma and ANG lesions. For example, both types of lesion had members of the genera *Prevotella*, *Peptostreptococcus*, and *Treponema* present [13], [14]. The healthy controls used in these studies were crucial for examining the role of *Fusobacterium necrophorum*, which was considered to be a culprit in the development of noma [5]. GESNOMA studies based on both low-throughput sequencing and microarray hybridization identified *Fusobacterium* to be as common in healthy controls as in lesions, suggesting its mere presence does not trigger noma [13], [14].

GESNOMA's low-throughput sequencing and microarray approaches led to discordant estimations of bacterial diversity between healthy and diseased sites: low-throughput sequencing suggested these communities were equally diverse [14], whereas hybridization of community DNA to microarrays indicated that noma had less diversity than healthy controls [13]. Though both of these technologies were powerful in their abilities to detect bacteria without imposing biases associated with culturing, they both lacked the ability to detect microbes of lower abundance, and the microarrays could not detect nucleic acids that were not included in the microarray design.

Our current study builds on these previous reports by using high-throughput sequencing to examine the bacterial communities in sixty samples from children in Niger. Improving the sequence coverage of these communities enabled the detection and quantification of bacteria that were less abundant and whose identities were not known in advance. We investigated whether the bacterial communities in lesions from noma differed from those in ANG lesions, compared to healthy gingival samples from the same children and gingival samples from healthy controls. Our goals were to begin cataloging the species present in individuals from the same villages who were healthy, suffering from noma, or suffering from ANG. This catalog will eventually serve as a powerful comparative tool for future longitudinal studies. We used this information to identify candidate bacterial species that are characteristic of acute noma lesions.

## Methods

### Study design and sample collection

As part of the GESNOMA study, subgingival fluid samples were collected with cotton points from noma, acute necrotizing gingivitis and control volunteers by nurses based in Zinder, Niger as described in [13], [14]. Samples were subsequently stored in guanidinium isothiocyanate medium (RLT buffer, Qiagen) at -80°C until processing.

### Ethics statement

This study is part of the GESNOMA project, an epidemiological data and sampling collection effort conducted between September 2001 and October 2006 in the Zinder area of Niger, Africa. Gingival fluids were collected by local nurses from children affected by ANG or noma along with gender matched controls. The children were between 6 months and 12 years of age at the time of sample collection. The study was carried out with the prior approval of the Niger Ministry of Health, the WHO noma programme in Niger, and the Swiss non-governmental organization “Sentinelles”, and complied with the Declaration of Helsinki. A brochure with an explanation of informed consent was prepared in French and translated orally by local staff to the children's parents or guardians. The parent or guardian of each child signed the consent form, usually by a cross and/or a fingerprint. The consent forms were all sent to the GESNOMA office in Geneva, Switzerland.

The Republic of Niger Ministry of Public Health approved the protocol in 2001. The Head of the Department of Studies and Programming of the Republic of Niger Ministry of Public Health approved the use of fingerprints as a signature in the case of illiteracy in advance. Additional information about the children in the study can be found in Table S1 and GESNOMA's epidemiological study [9].

### DNA extraction, amplification, and sequencing

DNA was extracted from stored samples using the DNeasy Blood & Tissue Kit (Qiagen). To facilitate cell lysis, samples were vortexed for 30 seconds with acid washed glass beads (Sigma #G1277, 212–300 µm). Then, 200 µl buffer AL was added and the protocol ‘Purification of Total DNA from Animal Blood’ from the’ DNeasy Blood & Tissue Handbook’ was followed. DNA was eluted in 30 µL of sterile water.

To amplify the V1–3 region of the bacterial 16S rRNA gene, 1 µL of DNA was combined with 0.5 µM of the forward primer 5′–gccttgccagcccgctcag*ac*GAGTTTGATCMTGGCTCAG–3′, 0.05 µM of an additional forward primer 5′–gccttgccagcccgctcag*ac*AGGGTTCGATTCTGGCTCAG–3′ and 0.5 µM barcoded reverse primer 5′–gcctccctcgcgccatcagNNNNNNNN*at*CCGCGRCTGCTGGCAC–3′ in 50 µl PrimeStar HS Premix (Takara). The structure of composite PCR primers is described in [16]. Amplification proceeded for 30 cycles of 98°C for 10 s, 56°C for 15 s, and 72°C for 1 min.

To amplify a segment of archaeal 16S rRNA gene, a two-step PCR was carried out to obtain sufficient material for sequencing. Initially, 2 µL of lysate was combined with 0.3 µM each of the forward 5′–CCGACGGTGAGRGRYGAA–3′ and reverse 5′–ACGGGCGGTGWGTRCAA–3′ primers in 25 µl PrimeStar HS Premix (Takara). Amplification proceeded for 33 cycles of 98°C for 10 s, 69°C for 15 s, and 72°C for 1 min. The second step used a 0.5 µl aliquot from the first reaction as template in a new PCR with 50 µL PrimeStar HS Premix (Takara) containing 0.5 µM each barcoded forward primer 5′–gcctccctcgcgccatcagNNNNNNNN*ta*CCGACGGTGAGRGRYGAA–3′ and reverse primer 5′–gccttgccagcccgctcag*ac*ACGGGCGGTGWGTRCAA–3′. Amplification proceeded for 28 cycles of 98°C for 10 s, 58°C for 15 s, and 72°C for 45 s.

For each sample, two identical PCRs were pooled and purified using the MinElute PCR purification kit (Qiagen), using 25 uL water for elution. Amplicon sizes and concentrations were estimated by running 1 µl of each amplicon on the Agilent 2100 Bioanalyzer using a DNA1000 lab chip. 100 ng of each purified amplicon was pooled and sequenced on a Genome Sequencer FLX system (Roche).

### Sequence read processing and analysis

**Figure 1:**
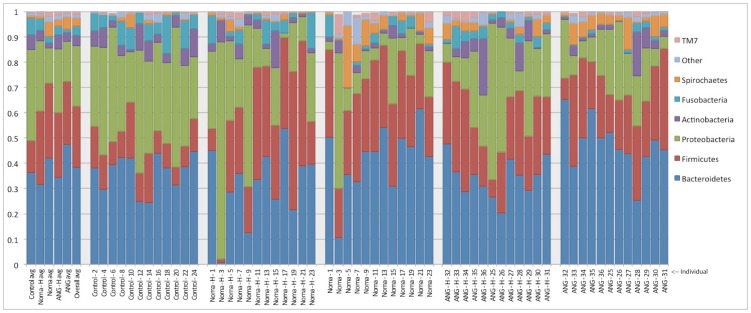
Abundance of Phyla across samples. The abundances of seven major bacterial phyla averaged across all samples of a given type (furthest panel on left), or for each individual sampled (five panels to the right). The y-axis indicates the proportion of each phylum.

The 91,114 sequence reads were trimmed using Qiime 1.2.0 [17]. Sequences were removed if they: contained any ambiguous bases (e.g., ‘N’), did not match the reverse primer or barcode, or had homopolymer stretches greater than 6 nucleotides. A large percentage of sequences were found to contain ambiguities at the ‘A’ in the second to last position at the 3′ end of the reverse primer; we allowed for only these ambiguities in order to retain more data. After trimming sequences to 200 bp, 71,715 sequences remained. These were binned by the 60 unique barcodes, representing the 60 samples that included 12 samples from each of the 5 health status groups. The sequences were clustered, using uclust implemented in Qiime [17], into OTUs using three similarity cut-offs: 90%, 97% and 99%. Chimeras were identified and removed using Chimera Slayer in Mothur [18]. Taxonomy was assigned to a representative sequence from each OTU using RDP in Qiime, and the output was used to create an OTU abundance table. Prior to OTU clustering, each read was also separately classified using RDP in Qiime. Results from these non-OTU based classifications are shown in Figure 1. A Bray-Curtis dissimilarity matrix based on the OTU abundance table was calculated using Primer 6.0 [19], and used as input for PERMANOVA [19] and Multi-Dimensional Scaling (MDS). A cytoscape network was built with the make_cytoscape_network.py command in Qiime; network edges were defined by the number of sequences in a particular OTU. The network was visualized with the organic layout in Cytoscape, so that the distance between nodes and edges in the network minimizes the force between them. Indicator Species Analysis (ISA) [20] was carried out using the labdsv package in R [21], [22]. The ISA score is calculated based on the relative abundance and frequency of each species by group [20]. Monte Carlo randomization procedures were then used to test the statistical significance of the highest indicator values. FDR values were estimated using the p.adjust function in R [22].

## Results

### Study design

Sixty samples collected from 36 children living in villages within a few hundred kilometers of Zinder, Niger were chosen for inclusion in the current study. Children were examined and sampled before treatment at the Sentinelles clinic by members of the GESNOMA study group [9], [14]. Gingival fluid from healthy and lesion sites of twelve children with noma and twelve children with ANG were included, along with twelve healthy controls [9], [14]. On average, noma lesions were sampled 11 days after their appearance. These sixty samples form five groups of twelve samples for comparison in this study: Noma lesions (N), healthy gingival samples from a noma affected mouth (NH), Acute Necrotizing Gingivitis lesions (ANG), healthy gingival samples from an Acute Necrotizing Gingivitis affected mouth (ANGH), and unaffected mouths from age, location and gender matched-controls (C).

The V1-3 segment of the bacterial 16S rRNA gene was amplified and sequenced (unidirectionally) using 454 pyrosequencing technology. We obtained 91,114 reads; after quality filtering 71,715 reads remained (see Methods), of which 11,438 were unique singletons. The distribution of sequence reads among the five health status categories was quite even, ranging from 13,520 to 15,993 reads per sample category (Table 1).

**Table 1:** Diversity comparisons between lesion and healthy sites*.

Diversity metric	Noma-healthy	Noma-lesion	p-value	ANG-healthy	ANG-lesion	p-value
**Chao1**	162 (57)	196 (43)	0.12	207 (52)	223 (38)	0.41
**Shannon**	4.78 (1.02)	5.36 (0.47)	0.08	5.51 (0.6)	5.48 (0.48)	0.87

Averages from individual samples (and standard deviations) are shown for each category. *Only includes sites from affected individuals, not healthy controls.

### Noma lesions carry a diverse bacterial community

To examine bacterial communities at different taxonomic levels, OTUs were calculated three separate times using 90%, 97%, and 99% sequence identity cut-offs. Here we present results from the 97% cutoff; results from the 90% and 99% cutoffs were similar (Table S4 and S5). OTUs shared between different health statuses varied from 262 to 358 (out of 1352 total; Table S4). OTUs from all three identity cutoffs were used to calculate the dissimilarities between communities using the Bray-Curtis metric. In all three cases, samples from sites similar in their health statuses harbored similar communities (i.e., lower dissimilarity values, in Table S5).

Bacterial diversity was measured by calculating Chao1 and Shannon diversity values for each health status group. Chao1 estimates diversity using only the number of OTUs present, whereas Shannon also considers the relative abundance of each OTU. Both diversity measures indicated that noma lesions support a greater diversity of bacteria than do healthy sites in diseased mouths; however, neither Chao1 or Shannon diversity measures differed significantly between wound and healthy sites (Table 1). This suggests that noma lesions are not dominated by a single pathogen that takes over the community; rather these lesions may develop due to a shift in the abundances of community members.

### Microbial communities differ between healthy and affected sites of diseased mouths

The distribution of phyla among the five sample categories revealed pronounced differences in their average abundances (Figure 1); we briefly summarize these differences here and present results from statistical analyses below. In healthy individuals, over 35% of sequences were assigned to *Proteobacteria*; in individuals with noma or ANG this dropped to fewer than 30%, regardless of whether a lesion or healthy site was sampled. Conversely, in healthy individuals *Firmicutes* accounted for less than 15% of reads, whereas in samples from both noma and ANG lesions this phylum accounted for over 20% of reads. Reads mapping to members of the phylum *Bacteroidetes* were more numerous in noma and ANG lesions, where they accounted for greater than 40% of the total, compared to less than 40% for healthy sites. *Spirochaetes* accounted for well over 5% of reads in lesion sites from both noma and ANG, which was noticeably higher than its average of ∼1% in healthy sites.

**Figure 2:**
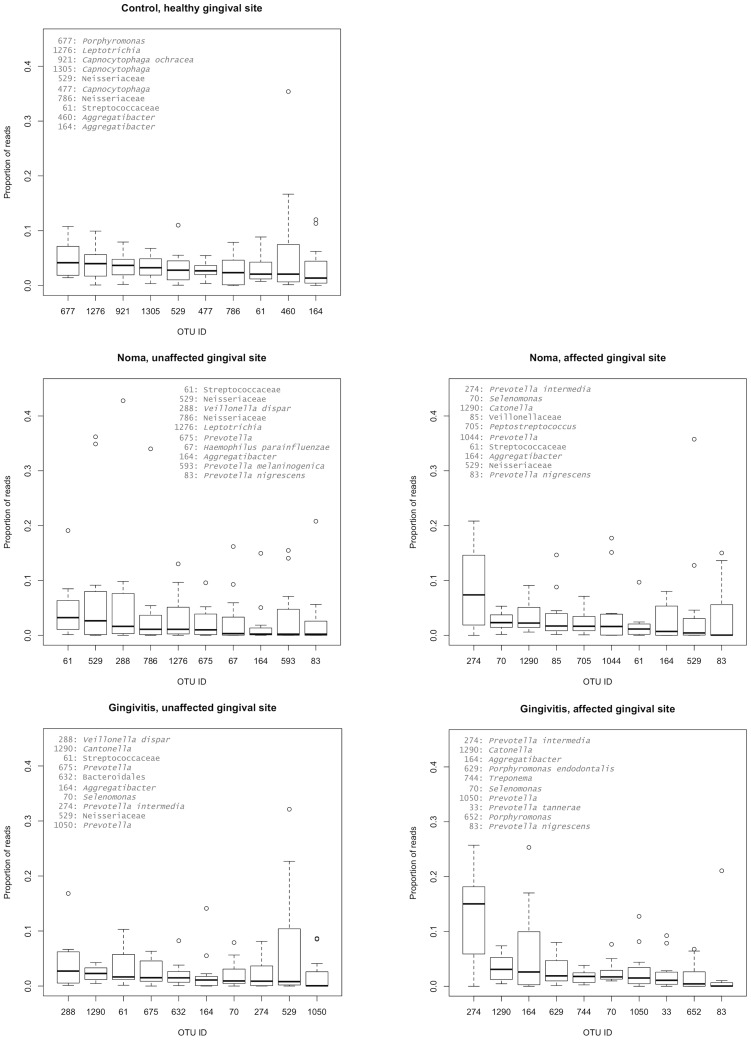
A box plot of the top 10 97% OTUs by group. For each sample type, the median and range of abundances of the most abundant OTUs are plotted. Circles indicate outliers (i.e., data points that are 1.5-fold lower or higher than the 25% and 75% quartiles, respectively).

The top ten most abundant 97% OTUs shown in Figure 2 reveal important differences in community structure between the lesion sites (noma and ANG) and healthy sites (from unaffected gingival fluid in noma and ANG volunteers, and the healthy volunteers). OTU 274, assigned to *Prevotella intermedia*, dominated wound sites, and had median values at least 3-fold higher than the next most abundant OTU. OTU 274 was also present in samples from healthy sites, though it was similar in abundance to other OTUs.

**Figure 3:**
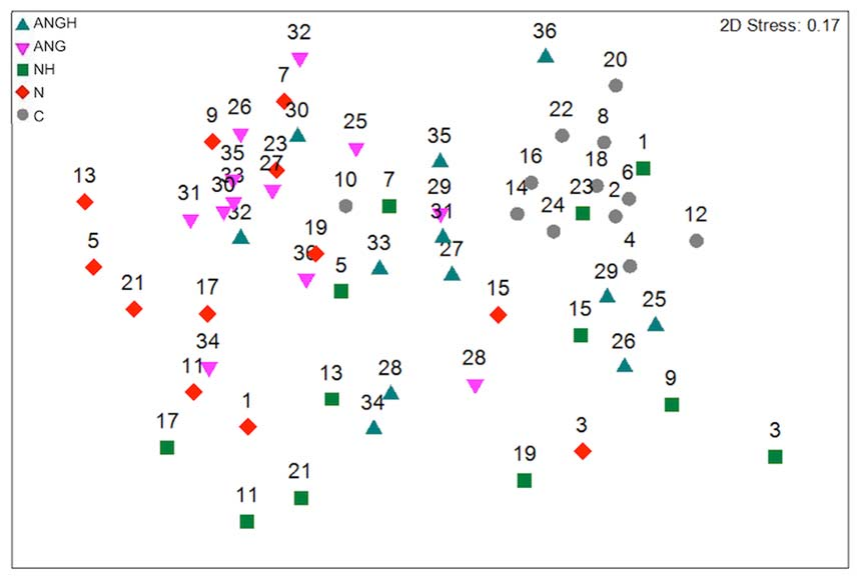
Multidimensional scaling (MDS) plot of Bray-Curtis dissimilarities using multi-dimensional scaling. Numbers correspond to individuals sampled, as reported in Figure 1.

Multi-dimensional scaling (MDS) of the Bray-Curtis dissimilarity matrix was used to visualize differences in community composition for each sample (Figure 3). The healthy controls had more similar microbial community composition, as reflected by their proximity to each other in the MDS plot. Samples from healthy sites in noma and ANG mouths spanned multivariate space between healthy individuals and samples from noma and ANG lesions. Lesions from noma and ANG affected mouths were variable in their community similarities, and although some samples were similar to healthy sites/controls, most of the lesion samples have unique community composition, as reflected by their distance from the other samples in the MDS projection. Samples from the same mouth did not typically cluster together, rather each sample clustered with samples from other mouths that had similar health statuses. To test whether groups significantly differed in their microbiota, we used PERMANOVA [23], a non-parametric multivariate analysis of variance, to test the null hypothesis that there were no differences between the health status groups (Table 2). Overall, PERMANOVA showed that the sample groups were significantly different from each other (*F*_999_ = 3.56, *P* = 0.001). Pairwise comparisons with p-values greater than 0.05 indicate that those two health status groups were not significantly different in the communities they support. We found that the communities from the lesion sites in noma and ANG mouths were more similar to each other (*F*_999_ = 1.22, *P* = 0.08) than they were to healthy sites in the same mouths or healthy mouths. This was also true for the healthy parts of the ANG and noma affected mouths (*F*_999_ = 1.08, *P* = 0.245). The control samples were most different from the ANG and N samples (Table 2). Similar results were obtained using Anosim [19] (Table S3).

**Table 2:** Comparisons of microbial community composition between the five health status groups.

Groups	t	P(perm)	P (FDR)*
**ANGH vs ANG**	1.68	0.003	0.005
**ANGH vs NH**	1.08	0.245	0.245
**ANGH vs N**	1.71	0.004	0.006
**ANGH vs C**	1.79	0.001	0.002
**ANG vs NH**	1.91	0.001	0.002
**ANG vs N**	1.22	0.08	0.089
**ANG vs C**	2.88	0.001	0.002
**NH vs N**	1.6	0.011	0.014
**NH vs C**	1.8	0.001	0.002
**N vs C**	2.86	0.001	0.002

Tests were performed with PERMANOVA using Bray-Curtis dissimilarity measures of OTU abundances (results from the 97% identity cutoff are shown here). The Pseudo-F statistic comparing all five groups is 3.56 with a permutation test P value of 0.001. The groups are defined as ANGH: Acute Necrotizing Gingivitis healthy site, ANG: Acute Necrotizing Gingivitis lesion site, C: Control, NH: Noma Healthy site, N: Noma lesion site. *P-values corrected for multiple comparisons using the False Discovery Rate. [42].

### Prevalence of candidates proposed in earlier studies

**Figure 4:**
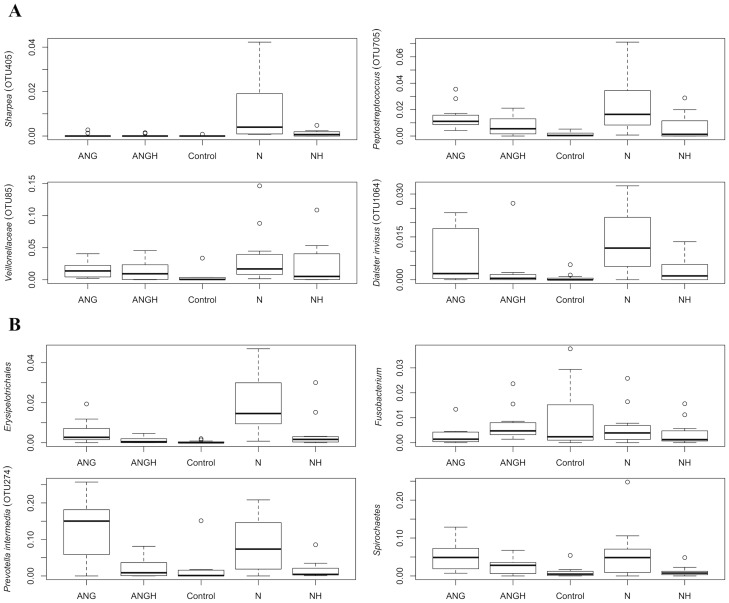
Box plots of abundances (proportion of total reads) for several bacterial taxa of interest. (A) The relative abundance of the top 4 97% OTUs that indicate the noma lesion samples are shown across the sample types (B) The relative abundances of several taxonomic groups of interest.

Attempts to understand the origin of noma have identified several bacterial species as candidate instigators of noma lesions. We report their abundances in each of the 5 health status categories as numbers of 97% OTUs in Table 3. Although a species in the genus *Fusobacterium* has been hypothesized to trigger noma, we found this phylum to be more abundant in the controls (Figure 4B). This pattern was also seen for the following hypothesized noma-triggering bacterial genera: *Porphyromonas, Capnocytophaga, Aggregatibacter* and *Streptococcus*. The opposite pattern, where abundances were higher in lesions than healthy controls, was observed for the phylum *Spirochaetes*, and the genera *Prevotella* and *Peptostreptococcus* (Figure 4A). Several organisms identified in noma infections by past studies were not found to be more abundant in noma in our dataset, including *Corynebacterium*, *Bacteroides*, and *Bacillus* (Table 3, [1]).

**Table 3:** Percentage of reads that correspond to bacterial groups suspected of triggering noma in the different sample groups, as determined by OTUs from the 97% cutoff.

Taxonomy	# 97% OTUs	Control	NH	Noma	ANGH	ANG
***Prevotella***	**248**	**8**	**20**	**32**	**17**	**33**
***Spirochaetes*** ** (includes ** ***Treponema*** **)**	**53**	**1.1**	**1.1**	**6.2**	**2.8**	**5.1**
*Porphyromonas*	50	10	4.3	4.4	6.3	9.7
*Aggregatibacter*	34	12.6	4.7	2.8	5.3	6.2
Streptococcaceae (includes *Streptococcus*)	25	3.7	6.3	2.7	4.0	1.7
***Peptostreptococcus***	**16**	**0.14**	**0.55**	**2.39**	**0.76**	**1.46**
*Flavobacteria*	57	14.1	5.3	0.6	6.2	1.7
*Fusobacterium*	7	0.87	0.30	0.61	0.75	0.31
*Capnocytophaga*	46	13.5	4.9	0.6	5.6	1.6
***Atopobium*** ** spp**	**3**	**0.02**	**0.03**	**0.08**	**0.01**	**0.11**
*Propionibacterium*	1	0.03	0.03	0*	0	0
*Stenotrophomonas*	1	0*	0*	0	0	0
*Corynebacterium*	9	1.5	0.3	0.2	1.0	0.3
*Bacteroides*	7	0.02	0.02	0.09	0.18	0.05
Bacillaceae (includes *Bacillus*)	3	0.03	0	0	0.03	0

Taxa indicated in bold are more abundant in noma or both noma and ANG samples compared to the control. Abbreviations are identical to those used in Table 2. *indicates 1 read obtained, which equals a percentage of <10^−3^.

### Indicator species analysis

To determine which taxa were significantly associated with noma lesion sites, we used indicator species analysis (ISA) [20]. The 12 noma lesion samples were compared against all other samples, including the healthy site samples from the same 12 individuals, and the ANG lesion site samples which had similar composition. ISA calculates the relative abundance and relative frequency of each species in each group, and compares this to a null distribution obtained by randomizing the taxon-sample relationships. These calculations were carried out separately with all three OTU sets (i.e., 90%, 97%, and 99%). We have reported results from the 97% OTUs here (Table 4); similar results were obtained with the 90% and 99% OTUs.

**Table 4:** Indicator species analysis comparing the noma lesion site samples (“Noma” in “Indicator of” column) with the other four health status groups (control, noma healthy site, ANG healthy site, and ANG lesion site; “Other” in “Indicator of” column).

OTU ID	Indicator of	Indicator value	P value	Taxon (Phylum; Class; Order; Family; Genus; Species)
**405**	**Noma**	**0.9626**	**0.001**	**Firmicutes; Erysipelotrichi; Erysipelotrichales; Coprobacillaceae; Sharpea**
921	Other	0.7947	0.001	Bacteroidetes; Flavobacteriia; Flavobacteriales; Flavobacteriaceae; Capnocytophaga; ochracea
288	Other	0.7558	0.019	Firmicutes; Clostridia; Clostridiales; Veillonellaceae; Veillonella; dispar
**705**	**Noma**	**0.7479**	**0.001**	**Firmicutes; Clostridia; Clostridiales; Peptostreptococcaceae; Peptostreptococcus**
477	Other	0.7108	0.002	Bacteroidetes; Flavobacteriia; Flavobacteriales; Flavobacteriaceae; Capnocytophaga
**85**	**Noma**	**0.7066**	**0.009**	**Firmicutes; Clostridia; Clostridiales; Veillonellaceae**
**1064**	**Noma**	**0.6984**	**0.003**	**Firmicutes; Clostridia; Clostridiales; Veillonellaceae; Dialister; invisus**
1305	Other	0.69	0.006	Bacteroidetes; Flavobacteriia; Flavobacteriales; Flavobacteriaceae; Capnocytophaga
675	Other	0.6836	0.016	Bacteroidetes; Bacteroidia; Bacteroidales; Prevotellaceae; Prevotella
285	Other	0.6815	0.003	Bacteroidetes; Flavobacteriia; Flavobacteriales; Flavobacteriaceae; Capnocytophaga; ochracea
**1060**	**Noma**	**0.679**	**0.003**	**Bacteroidetes; Bacteroidia; Bacteroidales; Prevotellaceae; Prevotella; melaninogenica**
1278	Other	0.6758	0.012	Proteobacteria; Betaproteobacteria; Burkholderiales; Burkholderiaceae; Lautropia
460	Other	0.6615	0.023	Proteobacteria; Gammaproteobacteria; Pasteurellales; Pasteurellaceae; Aggregatibacter
**1044**	**Noma**	**0.6531**	**0.002**	**Bacteroidetes; Bacteroidia; Bacteroidales; Prevotellaceae; Prevotella**
**667**	**Noma**	**0.6506**	**0.003**	**Bacteroidetes; Bacteroidia; Bacteroidales**
632	Other	0.6393	0.027	Bacteroidetes; Bacteroidia; Bacteroidales
786	Other	0.634	0.008	Proteobacteria; Betaproteobacteria; Neisseriales; Neisseriaceae
198	Other	0.632	0.038	Bacteroidetes; Bacteroidia; Bacteroidales; Porphyromonadaceae; Porphyromonas;
352	Other	0.6238	0.005	Firmicutes; Clostridia; Clostridiales; Veillonellaceae; Selenomonas; noxia
723	Other	0.5914	0.029	Proteobacteria; Gammaproteobacteria; Pasteurellales; Pasteurellaceae; Aggregatibacter
**1252**	**Noma**	**0.5895**	**0.038**	**Synergistetes; Synergistia; Synergistales; Dethiosulfovibrionaceae; TG5**
404	Other	0.5882	0.033	Firmicutes; Clostridia; Clostridiales; Veillonellaceae; Selenomonas
1291	Other	0.5882	0.029	Firmicutes; Clostridia; Clostridiales; Peptostreptococcaceae
**1013**	**Noma**	**0.5856**	**0.003**	**Firmicutes; Clostridia; Clostridiales; Clostridiaceae; Mogibacterium**
8	Other	0.5775	0.05	Bacteroidetes; Bacteroidia; Bacteroidales; Porphyromonadaceae; Porphyromonas
**535**	**Noma**	**0.5746**	**0.03**	**Firmicutes;** **Clostridia; Clostridiales; Veillonellaceae**
**881**	**Noma**	**0.5719**	**0.007**	**Firmicutes; Clostridia; Clostridiales; Veillonellaceae**
273	Other	0.5558	0.043	Actinobacteria; Actinobacteria; Actinomycetales; Corynebacteriaceae; Corynebacterium
**825**	**Noma**	**0.5556**	**0.045**	**Firmicutes; Clostridia; Clostridiales; Veillonellaceae; Selenomonas**
1022	Other	0.5466	0.022	Proteobacteria; Gammaproteobacteria; Pasteurellales; Pasteurellaceae; Haemophilus
1066	Other	0.5401	0.015	Bacteroidetes; Bacteroidia; Bacteroidales; Porphyromonadaceae; Porphyromonas
**770**	**Noma**	**0.5354**	**0.01**	**Bacteroidetes; Bacteroidia; Bacteroidales; Prevotellaceae; Prevotella; melaninogenica**
**1093**	**Noma**	**0.5354**	**0.011**	**Firmicutes; Erysipelotrichi; Erysipelotrichales; Erysipelotrichaceae; Bulleidia; moorei**
**496**	**Noma**	**0.53**	**0.036**	**Synergistetes; Synergistia; Synergistales; Dethiosulfovibrionaceae; TG5**
**1063**	**Noma**	**0.5272**	**0.007**	**Firmicutes; Clostridia; Clostridiales; Clostridiaceae**
973	Other	0.5219	0.025	Bacteroidetes; Flavobacteriia; Flavobacteriales; Flavobacteriaceae; Elizabethkingia
596	Other	0.5039	0.038	Actinobacteria; Actinobacteria; Actinomycetales; Propionibacteriaceae
**1142**	**Noma**	**0.4984**	**0.048**	**Spirochaetes; Spirochaetes; Spirochaetales; Spirochaetaceae; Treponema; amylovorum**
**756**	**Noma**	**0.4748**	**0.001**	**Firmicutes; Clostridia; Clostridiales; Lachnospiraceae; Oribacterium**
995	Other	0.4645	0.033	Bacteroidetes; Flavobacteriia; Flavobacteriales; Flavobacteriaceae; Capnocytophaga; ochracea
**866**	**Noma**	**0.4615**	**0.017**	**Firmicutes; Clostridia; Clostridiales; Ruminococcaceae**
1280	Other	0.4583	0.025	Firmicutes; Clostridia; Clostridiales; Lachnospiraceae; Johnsonella
**250**	**Noma**	**0.4569**	**0.002**	**Spirochaetes; Spirochaetes; Spirochaetales; Spirochaetaceae; Treponema**
**762**	**Noma**	**0.4523**	**0.028**	**Bacteroidetes; Bacteroidia; Bacteroidales; Paraprevotellaceae; Prevotella**
**438**	**Noma**	**0.443**	**0.005**	**Bacteroidetes; Bacteroidia; Bacteroidales; Prevotellaceae; Prevotella**
**463**	**Noma**	**0.4396**	**0.046**	**Firmicutes; Clostridia; Clostridiales; Veillonellaceae**
**39**	**Noma**	**0.4179**	**0.002**	**Firmicutes; Clostridia; Coriobacteriales; Coriobacteriaceae; Olsenella; uli**
711	Other	0.4089	0.033	Proteobacteria; Gammaproteobacteria; Pasteurellales; Pasteurellaceae; Haemophilus
**955**	**Noma**	**0.3974**	**0.026**	**Bacteroidetes; Bacteroidia; Bacteroidales**
**1205**	**Noma**	**0.391**	**0.03**	**Spirochaetes; Spirochaetes; Spirochaetales; Spirochaetaceae; Treponema**
**1261**	**Noma**	**0.3846**	**0.02**	**Bacteroidetes; Bacteroidia; Bacteroidales; Prevotellaceae; Prevotella**
943	Other	0.3542	0.043	Bacteroidetes; Bacteroidia; Bacteroidales
31	Other	0.3333	0.049	Proteobacteria; Betaproteobacteria; Rhodocyclales; Rhodocyclaceae; Propionivibrio
**820**	**Noma**	**0.3252**	**0.006**	**Spirochaetes; Spirochaetes; Spirochaetales; Spirochaetaceae; Treponema**
**659**	**Noma**	**0.303**	**0.039**	**Firmicutes; Clostridia; Clostridiales**
**355**	**Noma**	**0.2976**	**0.047**	**Fusobacteria; Fusobacteria; Fusobacteriales; Fusobacteriaceae; Fusobacterium**
**503**	**Noma**	**0.2963**	**0.02**	**Firmicutes; Erysipelotrichi; Erysipelotrichales; Erysipelotrichaceae; Bulleidia**
**1309**	**Noma**	**0.2963**	**0.022**	**Bacteroidetes; Bacteroidia; Bacteroidales; Prevotellaceae; Prevotella**
**200**	**Noma**	**0.25**	**0.01**	**Spirochaetes; Spirochaetes; Spirochaetales; Spirochaetaceae; Treponema**
**676**	**Noma**	**0.25**	**0.008**	**Bacteria**
**907**	**Noma**	**0.25**	**0.007**	**Bacteroidetes; Bacteroidia; Bacteroidales; Paraprevotellaceae; Prevotella**
**1124**	**Noma**	**0.25**	**0.006**	**Tenericutes; Mollicutes; Anaeroplasmatales; Anaeroplasmataceae**
**1317**	**Noma**	**0.2414**	**0.016**	**Firmicutes; Clostridia; Clostridiales; Clostridiaceae**
**471**	**Noma**	**0.24**	**0.011**	**Bacteroidetes; Bacteroidia; Bacteroidales; Prevotellaceae; Prevotella; melaninogenica**
**1154**	**Noma**	**0.24**	**0.013**	**Firmicutes; Clostridia; Clostridiales; Peptostreptococcaceae**
**1061**	**Noma**	**0.2353**	**0.012**	**Bacteroidetes; Bacteroidia; Bacteroidales**
**160**	**Noma**	**0.2319**	**0.018**	**Firmicutes; Clostridia; Clostridiales; Clostridiaceae; Peptoniphilus; asaccharolyticus**
**74**	**Noma**	**0.2299**	**0.039**	**Firmicutes; Clostridia; Coriobacteriales; Coriobacteriaceae; Atopobium**
**331**	**Noma**	**0.2222**	**0.029**	**TM7; TM7-3**
**768**	**Noma**	**0.2222**	**0.029**	**Bacteroidetes; Bacteroidia; Bacteroidales**
**1108**	**Noma**	**0.2143**	**0.047**	**Bacteroidetes; Bacteroidia; Bacteroidales; Bacteroidaceae; Bacteroides**
**1077**	**Noma**	**0.1875**	**0.047**	**Firmicutes; Clostridia; Clostridiales; Lachnospiraceae**
**94**	**Noma**	**0.1667**	**0.041**	**Firmicutes; Clostridia; Clostridiales; Clostridiaceae**
**249**	**Noma**	**0.1667**	**0.038**	**Bacteroidetes; Bacteroidia; Bacteroidales; Prevotellaceae; Prevotella; intermedia**
**580**	**Noma**	**0.1667**	**0.048**	**Firmicutes; Clostridia; Clostridiales; Peptostreptococcaceae; Peptostreptococcus**
**880**	**Noma**	**0.1667**	**0.034**	**Fusobacteria; Fusobacteria; Fusobacteriales; Leptotrichiaceae**
**931**	**Noma**	**0.1667**	**0.033**	**Bacteria**
**976**	**Noma**	**0.1667**	**0.042**	**Firmicutes**
**1113**	**Noma**	**0.1667**	**0.036**	**Bacteroidetes; Bacteroidia; Bacteroidales; Prevotellaceae; Prevotella**
**1150**	**Noma**	**0.1667**	**0.045**	**Bacteroidetes; Bacteroidia; Bacteroidales**
**1271**	**Noma**	**0.1667**	**0.038**	**Bacteroidetes; Bacteroidia; Bacteroidales; Prevotellaceae; Prevotella**
**736**	**Noma**	**0.1655**	**0.044**	**Proteobacteria; Gammaproteobacteria; Enterobacteriales; Enterobacteriaceae; Proteus**
**1318**	**Noma**	**0.1603**	**0.046**	**Bacteroidetes; Bacteroidia; Bacteroidales;** **Porphyromonadaceae; Paludibacter**
**614**	**Noma**	**0.16**	**0.046**	**Bacteroidetes; Bacteroidia; Bacteroidales; Prevotellaceae; Prevotella; intermedia**
**748**	**Noma**	**0.1569**	**0.038**	**Bacteroidetes; Bacteroidia; Bacteroidales; Prevotellaceae; Prevotella**

Indicator values were calculated using the abundance distribution of OTUs at the 97% identity cutoff. Boldface highlights the taxa that are indicators for noma. The abundance ranges for taxa with high indicator species values are shown in box plots in Figure 4A. Results with all groups in “Other” being treated separately are shown in Table S6.

The ISA identified several families that were significantly associated with noma lesions, which overall are consistent with the abundance plots shown in Figure 1. *Sharpea* (Family: Coprobacillaceae) was the taxon most discerning of noma, and its taxonomic Order Erysipelotrichales was consistently present as a significant indicator no matter which OTU cutoff was employed. Also associated with noma were several OTUs within the genera *Veillonella*, *Peptostreptococcus*, *Prevotella*, and *Treponema*. These genera are members of the *Firmicutes* phylum, except for the latter two, which are members of *Bacteroidetes* and *Spirochaetes*, respectively. Genera with OTUs indicative of non-noma sites included *Capnocytophaga* and *Aggregatibacter*, of the phyla *Flavobacteria* and *Proteobacteria*, respectively. Separate OTUs in the *Veillonella* and *Prevotella* genera were present in both noma and non-noma sites, indicating that species or strains in these genera differ in their ability to affect lesion development.

### Archaea

We specifically sought archaeal sequences in these samples, by repeating the sequencing using primers appropriate for the archaeal 16S rRNA gene. These sequences were analyzed with the same approach as sequences obtained using bacterial primers. Although all individuals produced amplicons with the archaeal primers, the vast majority of sequence reads mapped to bacterial taxa. Presumably this was due to little or no archaeal DNA being present, which then led to non-specific priming and amplification of bacterial templates. However, 15 individuals did produce some archaeal reads, ranging from 1 to 174 reads. Four individuals had 10 or more reads that corresponded to the archaeal phylum *Euryarchaeota*, with the highest read counts corresponding to the genus *Methanobrevibacter*.

### Network analysis

**Figure 5:**
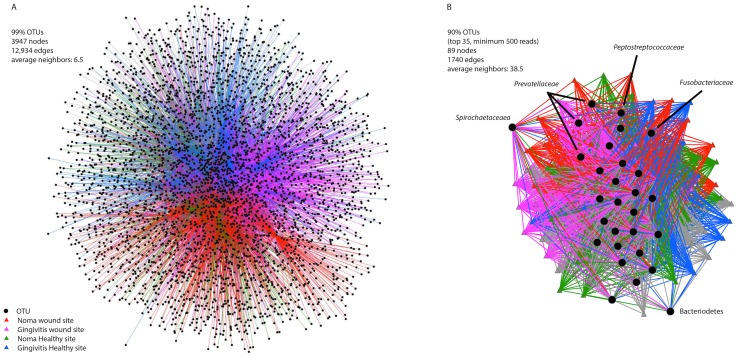
Network analysis of bacterial communities. The network in (A) was created using the 99% OTUs whereas (B) was created using the top 35 OTUs from the 90% cutoff dataset. The data in (B) are also shown as a barplot in Figure S1, and as a Venn diagram in Figure S2A.

To obtain a global view of the distribution of taxa across the health status groups, and at different levels of similarity, 99% OTUs and the top 35 OTUs with 90% identity were separately visualized in a Cytoscape network using the Qiime network building algorithm (Figure 5) [17], [24]. Individual samples and OTUs are displayed in the network, with lines connecting OTUs to the samples where they are present; shorter distances between the sample and OTU nodes reflect larger numbers of sequences from that OTU found in the connected sample. The OTUs that occur in the center of the network are more evenly distributed between all the samples, while those on the edges occur preferentially in certain samples. Spirochaetaceae and Prevotellaceae are both examples of taxa that were more prevalent in the lesion site samples, and they appeared tightly connected to disease site samples (Figure 5B). Overall, the courser grouping and inclusion of only the top 35 most abundant taxa led to a network where each node is connected to many more neighbors (38.5 neighbors in the 90% OTU network vs 6.5 in the 99% network). However strong clustering of sample site by color supports the statistically significant differences in community structure found using PERMANOVA (Table 2).

## Discussion

Noma is a devastating disease caused by an intricate interaction between socioeconomic conditions, immune system function and microbial factors. The goal of GESNOMA is to identify the microbial trigger of noma lesions, and here we move closer to that goal by thoroughly characterizing bacterial communities in healthy and diseased mouth sites. This is a formidable task, as accessing noma patients, particularly at the most critical time in development of their lesions, is challenging.

This work identified several groups of bacteria that were more abundant in and statistically associated with noma lesions. These included the genera *Treponema*, *Peptostreptococcus*, *Prevotella*, and *Sharpea*. There were multiple genera in the class *Clostridia* that were identified as indicators of noma, including: *Dialister*, *Peptostreptococcus, Mogibacterium, Selenomonas, Oribacterium, Olsenella, Peptoniphilus*, and *Atopobium*. One OTU classified as *Prevotella intermedia* (OTU 274) was the most abundant taxon in all samples from diseased individuals, but was much less abundant in healthy individuals. Within diseased individuals, this *Prevotella* OTU was ∼3-fold more abundant in lesion sites than healthy sites (Figure 2). GESNOMA studies based on low-throughput sequencing and microarrays agreed in their identification of *Peptostreptococcus* and *Prevotella* as being potential indicators of noma [9], [13], [14].

Because our information regarding these bacteria is limited to 16S rRNA gene community profiles, it is difficult to infer the potential pathogenicity of these organisms. Nevertheless it is useful to identify whether OTUs or their close relatives identified here are known to contribute to such destructive infections based on other clinical work [13], [14], [25], [26]. *Erysipelothrix rhusiopathiae* (of *Erysipelotrichales*) is capable of soft-tissue infections, and has been documented causing skin infections in farm workers and fisherman [26]–[28]. Furthermore, members of the bacterial genera *Peptostreptococcus*, *Veillonella*, *Prevotella*, and *Fusobacterium* are known to cause necrotizing infections of soft tissue [25], [26].

The presence and abundance of *Prevotella* in noma is consistent with other studies that used culture based methods [5], and DNA-based methods [13], [14]. *Prevotella* is also found in the oral microbiota of healthy individuals, but this does not preclude it from playing a role in triggering noma. Its abundance, and/or behavior in the context of adjacent community members, may be key. This is similar to the case of *Fusobacterium* that has been previously implicated in noma, and which we found to be abundant in healthy African controls from Niger. It is possible that alterations in community homeostasis that accompany early stages of disease enable *Prevotella* or *Fusobacterium* to dominate, which may hasten its evasion of the immune system, particularly a weak immune system. Such shifts in community activity may be detectable by examining changes in microbial gene expression or physiology during the development of noma lesions, a question suitable for an in-depth longitudinal study.

The noma lesion sites had slightly elevated Shannon and Chao1 diversity estimates compared to healthy sites from the same mouths, though the differences were not statistically significant (Table 1). This supports the idea that the dominance of an individual pathogen is not characteristic of noma lesions. In some human-associated microbial communities, such as those associated with pregnancy, gut inflammation, or obesity, disease associated samples undergo a reduction in richness [29]. However there are other instances where an increase in diversity is seen with disease, as is the case with oral infections (15). Furthermore, immunocompromised patients and those taking immunosuppressants after receiving an organ transplant harbor more diverse microbial and viral communities [30]. Thus, the way in which changes in bacterial diversity relate to the development of noma remains to be determined.

The work presented here supports the previously described similarities between communities in ANG and noma lesions, consistent with the hypothesis that ANG may be a precursor status to acute noma [31]. If this is indeed the case, screening susceptible children first for ANG would help to more quickly identify children at high-risk for noma. Although bacterial communities in each of these lesion types were distinguishable, they were also quite similar. For example, samples from lesions, whether they be from noma or ANG lesions, were more similar to each other than each was to a healthy site in the same mouth. The lack of total separation between the samples from each group using MDS reflects the fact that the communities of the two affected groups have similar compositions. This raises the possibility that the microbial trigger of noma is not a single bacterial species, but a multi-species consortium.

The expectation for an individual pathogen to dominate in an infection is a natural assumption supported by Koch's postulates. Recent insights into human-associated microbial communities, from culture-independent studies enabled by high-throughput sequencing, are changing how physicians and clinical microbiologists look at individual pathogens. A disturbed microbial community may be responsible for disease [11], particularly in cases where individuals do not have access to proper nutrition, leading to a weakened immune system. It is clear that human gingival fluid samples from all individuals, whether healthy or noma, share species. The samples taken from lesions of noma-affected children were not overwhelmingly dominated by a single invading pathogen or opportunist that was not found in the healthy controls. *Prevotella intermedia* OTU 274 was the most abundant bacterium in lesion sites; however diversity estimates indicate that the predominance of this taxon did not decrease overall bacterial diversity in noma or ANG lesions. This may explain why identifying a single noma lesion-triggering microorganism has been difficult. These data suggest that a single bacterial species may not be responsible for triggering noma. Additional potential triggers of noma include an unidentified fungal or viral species acting alone or together with the bacterial community, and alterations in bacterial physiology that prove detrimental to oral health.

While a bacterium may be identified as the cause of an infection using Koch's postulates, in most cases we lack knowledge of how the infection affects the structure of the microbial community [12]. Our survey of bacterial community structure in noma, ANG and healthy gingival samples from children in Niger has given us access to community composition data for a disorder that is likely to be polymicrobial. However, Koch's postulates were developed without considering an intricate polymicrobial community. Whether a particular microbe must comprise a certain fraction of a microbial community before physicians can determine its affect on clinical disease state, or whether the activity of less abundant microbes and their interactions with other community members more heavily influence disease state, is a current topic of debate [12], [32], [33]. In one study of children with Atopic Dermatitis, culture-independent 16S rRNA taxonomic profiles of skin swab samples, *Staphylococcus spp.* increased to 65±3.5% of the total population during a flare-up, and then returned to a statistically significant, much lower baseline (∼<20%) even without treatment [34]. Similarly, exacerbations in individuals with Cystic Fibrosis have been associated with an increased abundance of bacteria in the *Streptococcus milleri* group, an oral microbe that is not typically considered a pathogen in Cystic Fibrosis [35]. The distinction between infection and a healthy state may not be defined by the presence of the pathogenic microbe, but rather by the structure of the microbial community and their expressed genes, which is influenced by other complex forces such as the immune system. We must take this into account when we frame our hypotheses, and consider what it means for a microbe to trigger a noma lesion.

It is clear that noma is associated with a shift in the microbial community, and thus it should be possible to determine which species are significantly more abundant in noma samples, despite the limitations of a cross sectional study design with samples taken eleven days after the appearance of the noma wounds. Here we identified *Treponema, Prevotella, Peptostreptococcus* and *Sharpea* as indicators of noma lesions, consistent with previous observations (14). The expansion of *Prevotella intermedia* (OTU 274) in both the noma and ANG lesion sites is a significant finding made possible by the relative abundance data obtained through our 16S rDNA survey. Much like the community shifts that accompany dental caries and periodontal disease, changes in the microbial communities associated with ANG and noma may be driven by changes in the conditions in the oral cavity. In the case of dental caries, enamel-degrading acid is produced through the microbial fermentation of sugars, favoring acid-tolerant, tooth enamel demineralizing bacteria [36], [37]. Periodontal disease is associated with poor oral hygiene and may result from plaque-induced inflammation [38], [39]. Many of the bacteria identified in this study are known to ferment sugars under microaerophilic conditions [40]. Oral hygiene, microbial fermentation products and inflammation can all alter the presence and interactions of microbes. Such consequences of shifts in microbial community composition may be more serious in individuals with poor nutrition and a compromised immune system. Then, the community structure or activity shifts and detrimental gene expression or physiological changes come to dominate the oral ecosystem.

Longitudinal sampling could provide time-resolution to the data shown here, an important future goal for understanding the progression of noma. Watching the bacterial communities change as a noma lesion forms is critical; even our rigorous study design was only able to obtain samples from lesions that were on average eleven days old. Microbial metabolites produced by these oral microbes should also be monitored to provide additional information about the microbial activities that affect the development of noma. This in turn would help to inform antibiotic treatments, and pave the way for the design of novel therapies.

In summary, our high-throughput sequencing approach identified bacterial taxa consistent with previous culture-based and DNA-based studies. These results are also consistent with the hypothesis that ANG is a first step towards the development of noma; however this must be verified through additional study of these two diseases. These results also suggest that noma lesions have a greater diversity of bacteria than healthy sites in control and noma individuals. Interestingly, these lesions also had a greater abundance of anaerobic bacteria capable of fermentation (e.g. *Prevotella*). The inclusion of matched healthy controls has complicated the idea that the presence of a single bacterial species can trigger noma. For example, *Fusobacterium* was previously thought to be a noma trigger, though we found it to be abundant in healthy controls. These results indicate that noma should be viewed and treated as a polymicrobial infection, and that future longitudinal studies will be key to identifying the underlying microbial processes that lead to noma.

### Data access

The 16S rRNA gene amplicon sequence data are available at the MG-RAST website (http://metagenomics.anl.gov/) with ID# 4585252.3. The fasta sequence file available there contains output from the ‘split.libraries.py’ command in Qiime; each fasta header includes information identifying which sample the sequence was derived from. The samples are further described, using the same identifiers found in the fasta file headers, in Table S1.

## Supporting Information

Checklist S1 STROBE checklist.
(DOC)

Figure S1 Top 35 97% OTU distribution across sample categories. The X axis is labeled with the OTU number, and in parentheses is the number of reads corresponding to that OTU in the entire dataset. OTU identities are shown in Table S2.
(TIF)

Figure S2 Venn diagram showing the overlap of studied communities (A) and taxa (B, C, & D). Categories are labeled as NH: Noma Healthy; N: Noma; C: Control; GH: Gingivitis Healthy; G: Gingivitis.
(TIF)

Table S1 Clinical information about patients included in the study. Further details can be found in Baratti-Mayer et al. (2013) [9].
(DOCX)

Table S2 Taxonomy of the top 35 OTUs at a 97% identity cutoff as determined using Qiime with the greengenes alignment. The distribution of these OTUs can be seen in Figure S1.
(DOCX)

Table S3 Analysis of similarity (Anosim) pairwise results with the five categories of samples. The global sample statistic (Global R) is 0.341, below the cutoff of 0.4 which is described as significant [41]. The significance level of sample statistic is 0.01% with 9999 permutations. The number of permuted statistics greater than or equal to Global R is 0. The categories are 1. Noma Healthy site, 2. Noma wounded site, 3. Control, 4. Acute necrotizing gingivitis healthy site, 5. Acute necrotizing gingivitis wound site.
(DOCX)

Table S4 Shared OTUs at 97% (on top, in bold) and 99% clustering cut-offs.
(DOCX)

Table S5 Pairwise sample dissimilarities, calculated using the Bray-Curtis measure, at different levels of OTU identity cut-off.
(DOCX)

Table S6 Indicator species analysis with comparison of different sample groups. Red indicates enrichment in a disease state (Noma or ANG) and black indicates enrichment in the control or non-disease state.
(DOCX)
