# Supplementary material for: Noma Affected Children from Niger Have Distinct Oral Microbial Communities Based on High-Throughput Sequencing of 16S rRNA Gene Fragments
Source: PLoS Negl Trop Dis. 2014 Dec 4;8(12):e3240. doi: 10.1371/journal.pntd.0003240 (PMC4256271; doi:10.1371/journal.pntd.0003240)
Supplement: Checklist S1 — STROBE checklist. (DOC) [file pntd.0003240.s001.doc]

**Checklist S1**: STROBE Statement—Checklist of items that should be included in reports of ***cross-sectional studies*** Responses highlighted in yellow

|  | Item No | Recommendation |
| --- | --- | --- |
| **Title and abstract** | 1 | (*a*) Indicate the study’s design with a commonly used term in the title or the abstract |
|  |  | (*b*) Provide in the abstract an informative and balanced summary of what was done and what was found (a) Line 40, (b) Lines 39-54 |
| Introduction | | |
| Background/rationale | 2 | Explain the scientific background and rationale for the investigation being reported Lines 72-107 |
| Objectives | 3 | State specific objectives, including any prespecified hypotheses Lines 150-158 |
| Methods | | |
| Study design | 4 | Present key elements of study design early in the paper Lines 39-42 and 161-179 |
| Setting | 5 | Describe the setting, locations, and relevant dates, including periods of recruitment, exposure, follow-up, and data collection Lines 166-179; Supplemental Table 1 |
| Participants | 6 | (*a*) Give the eligibility criteria, and the sources and methods of selection of participants lines 165-178, 231-240, also in references 9, 13 and 14 from same study |
| Variables | 7 | Clearly define all outcomes, exposures, predictors, potential confounders, and effect modifiers. Give diagnostic criteria, if applicable Table S1, also see references 9 and 14 |
| Data sources/ measurement | 8* | For each variable of interest, give sources of data and details of methods of assessment (measurement). Describe comparability of assessment methods if there is more than one group Lines 180-228 |
| Bias | 9 | Describe any efforts to address potential sources of bias lines 100-108, 145-147 and 459-461 |
| Study size | 10 | Explain how the study size was arrived at – lines 231-240 |
| Quantitative variables | 11 | Explain how quantitative variables were handled in the analyses. If applicable, describe which groupings were chosen and why Lines 208-228 |
| Statistical methods | 12 | (*a*) Describe all statistical methods, including those used to control for confounding Lines 208-228 |
|  |  | (*b*) Describe any methods used to examine subgroups and interactions Not applicable |
|  |  | (*c*) Explain how missing data were addressed Not applicable |
|  |  | (*d*) If applicable, describe analytical methods taking account of sampling strategy Not applicable |
|  |  | (*e*) Describe any sensitivity analyses Not applicable |
| Results | | |
| Participants | 13* | (a) Report numbers of individuals at each stage of study—eg numbers potentially eligible, examined for eligibility, confirmed eligible, included in the study, completing follow-up, and analysed Lines 233-240 |
|  |  | (b) Give reasons for non-participation at each stage Not applicable |
|  |  | (c) Consider use of a flow diagram Not necessary |
| Descriptive data | 14* | (a) Give characteristics of study participants (eg demographic, clinical, social) and information on exposures and potential confounders Lines 160-178 and Table S1 |
|  |  | (b) Indicate number of participants with missing data for each variable of interest Not applicable |
| Outcome data | 15* | Report numbers of outcome events or summary measures Lines 259-261 and Table 1; Lines 266-275 and Figure 1 |
| Main results | 16 | (*a*) Give unadjusted estimates and, if applicable, confounder-adjusted estimates and their precision (eg, 95% confidence interval). Make clear which confounders were adjusted for and why they were included Table 1 |
|  |  | (*b*) Report category boundaries when continuous variables were categorized Not applicable |
|  |  | (*c*) If relevant, consider translating estimates of relative risk into absolute risk for a meaningful time period Not applicable |
| Other analyses | 17 | Report other analyses done—eg analyses of subgroups and interactions, and sensitivity analyses Lines 284-302; Lines 317-335; Lines 348-360 |
| Discussion | | |
| Key results | 18 | Summarise key results with reference to study objectives Lines 371-379 |
| Limitations | 19 | Discuss limitations of the study, taking into account sources of potential bias or imprecision. Discuss both direction and magnitude of any potential bias Lines 381-382; Lines 456-459 |
| Interpretation | 20 | Give a cautious overall interpretation of results considering objectives, limitations, multiplicity of analyses, results from similar studies, and other relevant evidence Lines 474-492 |
| Generalisability | 21 | Discuss the generalisability (external validity) of the study results Lines 411-414 |
| Other information | | |
| Funding | 22 | Give the source of funding and the role of the funders for the present study and, if applicable, for the original study on which the present article is based Hirzel Foundation |

*Give information separately for exposed and unexposed groups.

**Note:** An Explanation and Elaboration article discusses each checklist item and gives methodological background and published examples of transparent reporting. The STROBE checklist is best used in conjunction with this article (freely available on the Web sites of PLoS Medicine at http://www.plosmedicine.org/, Annals of Internal Medicine at http://www.annals.org/, and Epidemiology at http://www.epidem.com/). Information on the STROBE Initiative is available at www.strobe-statement.org.
