# Supplementary figures and images for: Noma Affected Children from Niger Have Distinct Oral Microbial Communities Based on High-Throughput Sequencing of 16S rRNA Gene Fragments
Source: PLoS Negl Trop Dis. 2014 Dec 4;8(12):e3240. doi: 10.1371/journal.pntd.0003240 (PMC4256271; doi:10.1371/journal.pntd.0003240)

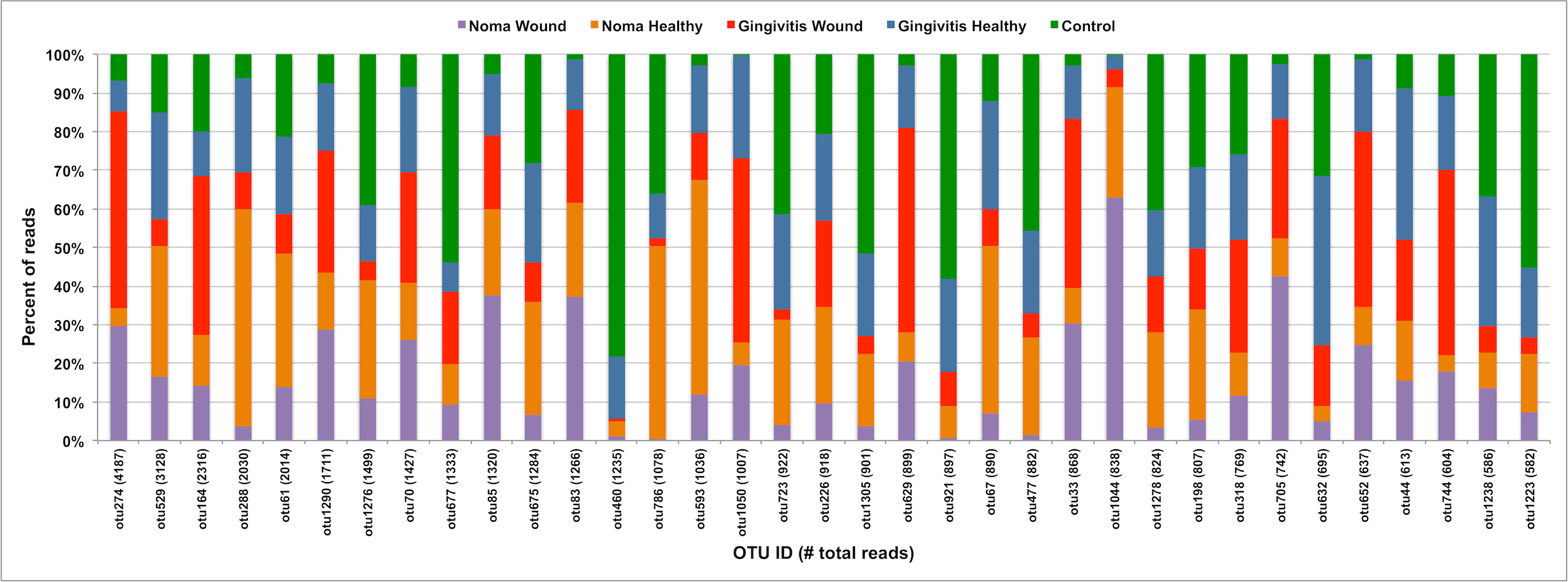

Supplement: Figure S1 — Top 35 97% OTU distribution across sample categories. The X axis is labeled with the OTU number, and in parentheses is the number of reads corresponding to that OTU in the entire dataset. OTU identities are shown in Table S2. (TIF) [file pntd.0003240.s002.tif]

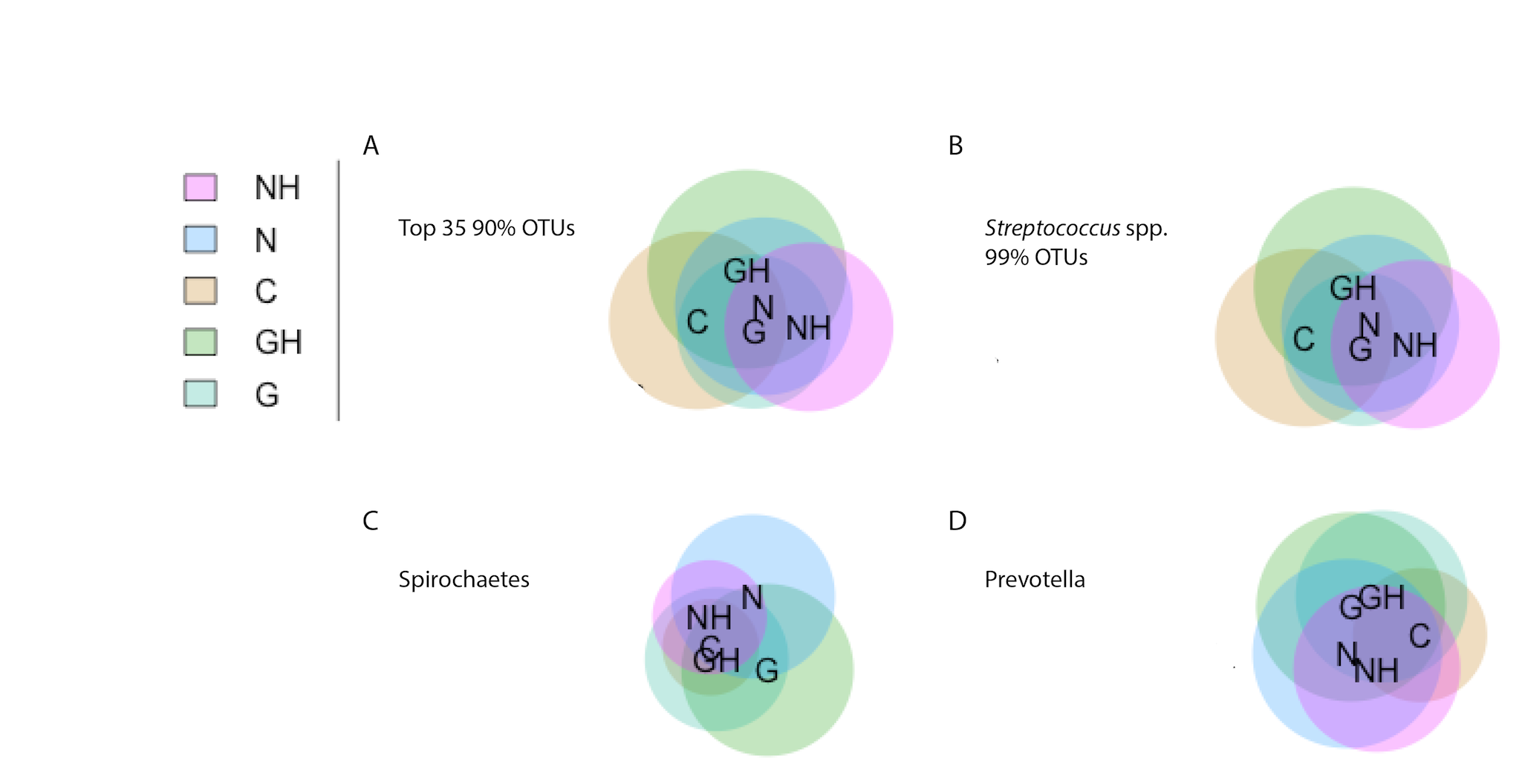

Supplement: Figure S2 — Venn diagram showing the overlap of studied communities (A) and taxa (B, C, & D). Categories are labeled as NH: Noma Healthy; N: Noma; C: Control; GH: Gingivitis Healthy; G: Gingivitis. (TIF) [file pntd.0003240.s003.tif]
