# Supplementary material for: Noma Affected Children from Niger Have Distinct Oral Microbial Communities Based on High-Throughput Sequencing of 16S rRNA Gene Fragments
Source: PLoS Negl Trop Dis. 2014 Dec 4;8(12):e3240. doi: 10.1371/journal.pntd.0003240 (PMC4256271; doi:10.1371/journal.pntd.0003240)
Supplement: Table S1 — Clinical information about patients included in the study. Further details can be found in Baratti-Mayer et al. (2013) [9]. (DOCX) [file pntd.0003240.s004.docx]

**Table S1.** Clinical information about patients included in the study. Further details can be found in: Baratti-Mayer D, Pittet B, Montandon D, Bolivar I, Bornand J-E, et al. (2003) Noma: an "infectious" disease of unknown aetiology. The Lancet infectious diseases 3: 419-431.

| # | Fasta ID | Age  (years) | Gender | Village | km from  Zinder | Father's  Occupation | Health  Status |
| --- | --- | --- | --- | --- | --- | --- | --- |
| 1 | 48, 49 | 6 | M | Bakin Birdji | 55 | revendeur | Noma |
| 2 | 50 | 6 | M | Bakin Birdji | 55 | agriculteur | Control |
| 3 | 51, 52 | 2 | F | Zinder (Guindawa) | 2 | marabout | Noma |
| 4 | 53 | 3 | M | Zinder (Galadima) | 2 | gardien | Control |
| 5 | 54, 55 | 3 | M | Rabi | 43 | agriculteur | Noma |
| 6 | 56 | 3 | F | Rabi | 43 | agriculteur | Control |
| 7 | 57, 58 | 4 | F | Magagi | 170 | agriculteur | Noma |
| 8 | 59 | 4 | F | Magagi | 170 | agriculteur | Control |
| 9 | 60, 61 | 8 | F | Chadawanna | 47 | agriculteur | Noma |
| 10 | 62 | 8 | F | Chadawanna | 47 | revendeur | Control |
| 11 | 63, 64 | 2 | M | Nakoni Haïdo | 389 | agriculteur | Noma |
| 12 | 65 | 2 | M | Nakoni Haïdo | 389 | agriculteur | Control |
| 13 | 66, 67 | 2 | F | Jataka | 362 | revendeur | Noma |
| 14 | 68 | 3 | F | Jataka | 362 | agriculteur | Control |
| 15 | 69, 70 | 4 | F | ? |  | chauffeur | Noma |
| 16 | 71 |  |  | ? |  |  | Control |
| 17 | 72, 73 | 3 | M | Gassaou (Guidan Idder) | 542 | agriculteur | Noma |
| 18 | 74 | 3 | F | Gassaou (Guidan Idder) | 542 | agriculteur | Control |
| 19 | 75, 76 | 3 | F | Gassaou (Guidan Idder) | 542 | agriculteur | Noma |
| 20 | 77 | 3 | M | Gassaou (Guidan Idder) | 542 | agriculteur | Control |
| 21 | 78, 79 | 3 | F | Bani Mayantchi | 30 | agriculteur | Noma |
| 22 | 80 | 5 | M | Bani Mayantchi | 30 | agriculteur | Control |
| 23 | 81, 82 | 8 | F | Bani Mayantchi | 30 | agriculteur | Noma |
| 24 | 83 | 8 | M | Bani Mayantchi | 30 | agriculteur | Control |
| 25 | 84, 85 | 6 | M | Zinder (Galadima) | 1 | transposteur | Gingivitis |
| 26 | 86, 87 | 7 | M | Zinder (Karakara) | 1 | DCD | Gingivitis |
| 27 | 88,89 | 3 | M | Zinder (Garin Malam) | 1 | revendeur | Gingivitis |
| 28 | 90, 91 | 2 | M | Zinder (Djaguindi) | 1 | revendeur | Gingivitis |
| 29 | 92, 94 | 3 | M | Zinder (Tabkin Tchakoua) | 1 | revendeur | Gingivitis |
| 30 | 95, 96 | 6 | F | Zinder (Angala Birni) | 1 | blanchisseur | Gingivitis |
| 31 | 97, 98 | 4 | F | Zinder (Garin Malam) | 2 | marabout | Gingivitis |
| 32 | 142, 143 | 5 | F | Tachalawa | 415 | revendeur | Gingivitis |
| 33 | 144, 145 | 6 | M | Katohou | 10 | marabout | Gingivitis |
| 34 | 148, 149 | 3 | F | Zinder (Birni Tirquet) | 3 | boucher | Gingivitis |
| 35 | 150, 151 | 9 | F | Zinder (Charé Zamna) | 1 | retraité | Gingivitis |
| 36 | 152, 153 | 5 | F | Zinder (Makarahouta) | 1 | agriculteur | Gingivitis |
