# Supplementary material for: Noma Affected Children from Niger Have Distinct Oral Microbial Communities Based on High-Throughput Sequencing of 16S rRNA Gene Fragments
Source: PLoS Negl Trop Dis. 2014 Dec 4;8(12):e3240. doi: 10.1371/journal.pntd.0003240 (PMC4256271; doi:10.1371/journal.pntd.0003240)
Supplement: Table S2 — Taxonomy of the top 35 OTUs at a 97% identity cutoff as determined using Qiime with the greengenes alignment. The distribution of these OTUs can be seen in Figure S1. (DOCX) [file pntd.0003240.s005.docx]

**Table S2.** Taxonomy of the top 35 OTUs at a 97% identity cutoff as determined using Qiime with the greengenes alignment. The distribution of these OTUs can be seen in Supplemental Figure 1.

| OTU ID | NH | Control | N | ANG | ANGH | taxonomy |
| --- | --- | --- | --- | --- | --- | --- |
| 274 | 198 | 284 | 1241 | 2137 | 327 | k__Bacteria; p__Bacteroidetes; c__Bacteroidia; o__Bacteroidales; f__Prevotellaceae; g__Prevotella; s__intermedia |
| 529 | 1055 | 473 | 517 | 217 | 866 | k__Bacteria; p__Proteobacteria; c__Betaproteobacteria; o__Neisseriales; f__Neisseriaceae; g__; s__ |
| 164 | 309 | 462 | 326 | 949 | 270 | k__Bacteria; p__Proteobacteria; c__Gammaproteobacteria; o__Pasteurellales; f__Pasteurellaceae; g__Aggregatibacter |
| 288 | 1142 | 123 | 77 | 191 | 497 | k__Bacteria; p__Firmicutes; c__Clostridia; o__Clostridiales; f__Veillonellaceae; g__Veillonella; s__dispar |
| 61 | 697 | 429 | 277 | 207 | 404 | k__Bacteria; p__Firmicutes; c__Bacilli; o__Lactobacillales; f__Streptococcaceae; g__; s__ |
| 1290 | 254 | 127 | 491 | 541 | 298 | k__Bacteria; p__Firmicutes; c__Clostridia; o__Clostridiales; f__Lachnospiraceae; g__Catonella; s__ |
| 1276 | 455 | 587 | 166 | 76 | 215 | k__Bacteria; p__Fusobacteria; c__Fusobacteria; o__Fusobacteriales; f__Leptotrichiaceae; g__Leptotrichia; s__ |
| 70 | 212 | 120 | 371 | 408 | 316 | k__Bacteria; p__Firmicutes; c__Clostridia; o__Clostridiales; f__Veillonellaceae; g__Selenomonas; s__ |
| 677 | 141 | 716 | 122 | 253 | 101 | k__Bacteria; p__Bacteroidetes; c__Bacteroidia; o__Bacteroidales; f__Porphyromonadaceae; g__Porphyromonas; s__ |
| 85 | 295 | 68 | 496 | 251 | 210 | k__Bacteria; p__Firmicutes; c__Clostridia; o__Clostridiales; f__Veillonellaceae; g__; s__ |
| 675 | 378 | 362 | 84 | 130 | 330 | k__Bacteria; p__Bacteroidetes; c__Bacteroidia; o__Bacteroidales; f__Prevotellaceae; g__Prevotella; s__ |
| 83 | 310 | 17 | 470 | 302 | 167 | k__Bacteria; p__Bacteroidetes; c__Bacteroidia; o__Bacteroidales; f__Prevotellaceae; g__Prevotella; s__nigrescens |
| 460 | 48 | 967 | 12 | 8 | 200 | k__Bacteria; p__Proteobacteria; c__Gammaproteobacteria; o__Pasteurellales; f__Pasteurellaceae; g__Aggregatibacter |
| 786 | 540 | 388 | 5 | 19 | 126 | k__Bacteria; p__Proteobacteria; c__Betaproteobacteria; o__Neisseriales; f__Neisseriaceae; g__; s__ |
| 593 | 575 | 30 | 123 | 127 | 181 | k__Bacteria; p__Bacteroidetes; c__Bacteroidia; o__Bacteroidales; f__Prevotellaceae; g__Prevotella; s__melaninogenica |
| 1050 | 60 | 2 | 196 | 480 | 269 | k__Bacteria; p__Bacteroidetes; c__Bacteroidia; o__Bacteroidales; f__Prevotellaceae; g__Prevotella; s__ |
| 723 | 254 | 381 | 36 | 24 | 227 | k__Bacteria; p__Proteobacteria; c__Gammaproteobacteria; o__Pasteurellales; f__Pasteurellaceae; g__Aggregatibacter; s__ |
| 226 | 232 | 189 | 87 | 205 | 205 | k__Bacteria; p__Actinobacteria; c__Actinobacteria; o__Actinomycetales; f__Actinomycetaceae; g__Parascardovia; s__ |
| 1305 | 169 | 466 | 32 | 43 | 191 | k__Bacteria; p__Bacteroidetes; c__Flavobacteriia; o__Flavobacteriales; f__Flavobacteriaceae; g__Capnocytophaga; s__ |
| 629 | 68 | 27 | 183 | 478 | 143 | k__Bacteria; p__Bacteroidetes; c__Bacteroidia; o__Bacteroidales; f__Porphyromonadaceae; g__Porphyromonas; s__endodontalis |
| 921 | 76 | 522 | 5 | 80 | 214 | k__Bacteria; p__Bacteroidetes; c__Flavobacteriia; o__Flavobacteriales; f__Flavobacteriaceae; g__Capnocytophaga; s__ochracea |
| 67 | 388 | 107 | 61 | 85 | 249 | k__Bacteria; p__Proteobacteria; c__Gammaproteobacteria; o__Pasteurellales; f__Pasteurellaceae; g__Haemophilus; s__parainfluenzae |
| 477 | 223 | 404 | 12 | 56 | 187 | k__Bacteria; p__Bacteroidetes; c__Flavobacteriia; o__Flavobacteriales; f__Flavobacteriaceae; g__Capnocytophaga; s__ |
| 33 | 80 | 26 | 263 | 379 | 120 | k__Bacteria; p__Bacteroidetes; c__Bacteroidia; o__Bacteroidales; f__[Paraprevotellaceae]; g__[Prevotella]; s__tannerae |
| 1044 | 241 | 1 | 526 | 39 | 31 | k__Bacteria; p__Bacteroidetes; c__Bacteroidia; o__Bacteroidales; f__Prevotellaceae; g__Prevotella; s__ |
| 1278 | 204 | 332 | 28 | 117 | 143 | k__Bacteria; p__Proteobacteria; c__Betaproteobacteria; o__Burkholderiales; f__Burkholderiaceae; g__Lautropia; s__ |
| 198 | 231 | 236 | 42 | 128 | 170 | k__Bacteria; p__Bacteroidetes; c__Bacteroidia; o__Bacteroidales; f__Porphyromonadaceae; g__Porphyromonas; s__ |
| 318 | 87 | 198 | 88 | 225 | 171 | k__Bacteria; p__Proteobacteria; c__Epsilonproteobacteria; o__Campylobacterales; f__Campylobacteraceae; g__Campylobacter |
| 705 | 72 | 20 | 316 | 230 | 104 | k__Bacteria; p__Firmicutes; c__Clostridia; o__Clostridiales; f__Peptostreptococcaceae; g__Peptostreptococcus |
| 632 | 28 | 218 | 34 | 109 | 306 | k__Bacteria; p__Bacteroidetes; c__Bacteroidia; o__Bacteroidales |
| 652 | 64 | 8 | 157 | 289 | 119 | k__Bacteria; p__Bacteroidetes; c__Bacteroidia; o__Bacteroidales; f__Porphyromonadaceae; g__Porphyromonas; s__ |
| 44 | 94 | 54 | 95 | 130 | 240 | k__Bacteria; p__Firmicutes; c__Clostridia; o__Clostridiales; f__Lachnospiraceae; g__; s__ |
| 744 | 26 | 65 | 107 | 291 | 115 | k__Bacteria; p__Spirochaetes; c__Spirochaetes; o__Spirochaetales; f__Spirochaetaceae; g__Treponema; s__ |
| 1238 | 54 | 216 | 79 | 41 | 196 | k__Bacteria; p__Proteobacteria; c__Epsilonproteobacteria; o__Campylobacterales; f__Campylobacteraceae; g__Campylobacter; s__ |
| 1223 | 88 | 321 | 42 | 26 | 105 | k__Bacteria; p__Proteobacteria; c__Betaproteobacteria; o__Neisseriales; f__Neisseriaceae |
