# Supplementary material for: Noma Affected Children from Niger Have Distinct Oral Microbial Communities Based on High-Throughput Sequencing of 16S rRNA Gene Fragments
Source: PLoS Negl Trop Dis. 2014 Dec 4;8(12):e3240. doi: 10.1371/journal.pntd.0003240 (PMC4256271; doi:10.1371/journal.pntd.0003240)
Supplement: Table S3 — Analysis of similarity (Anosim) pairwise results with the five categories of samples. The global sample statistic (Global R) is 0.341, below the cutoff of 0.4 which is described as significant [41]. The significance level of sample statistic is 0.01% with 9999 permutations. The number of permuted statistics greater than or equal to Global R is 0. The categories are 1. Noma Healthy site, 2. Noma wounded site, 3. Control, 4. Acute necrotizing gingivitis healthy site, 5. Acute necrotizing gingivitis wound site. (DOCX) [file pntd.0003240.s006.docx]

**Table S3:** Analysis of similarity (Anosim) pairwise results with the five categories of samples. The global sample statistic (Global R) is 0.341, below the cutoff of 0.4 which is described as significant (Clarke, 1993). The significance level of sample statistic is 0.01% with 9999 permutations. The number of permuted statistics greater than or equal to Global R is 0. The categories are 1. Noma Healthy site, 2. Noma wounded site, 3. Control, 4. Acute necrotizing gingivitis healthy site, 5. Acute necrotizing gingivitis wound site.

*Pairwise Tests*

R Significance Possible Actual Number >=

Groups Statistic Level % Permutations Permutations Observed

4, 5 0.258 0.4 1352078 9999 35

4, 1 0.042 20 1352078 9999 1995

4, 2 0.287 0.2 1352078 9999 16

4, 3 0.309 0.02 1352078 9999 1

5, 1 0.371 0.01 1352078 9999 0

5, 2 0.084 5.8 1352078 9999 579

5, 3 0.741 0.01 1352078 9999 0

1, 2 0.239 0.5 1352078 9999 52

1, 3 0.29 0.03 1352078 9999 2

2, 3 0.741 0.01 1352078 9999 0

Clarke, K. R. (1993) Non-parametric multivariate analyses of changes in community structure. Australian Journal of Ecology, v. 18, n. 1, p. 117–143.
