# Supplementary material for: Noma Affected Children from Niger Have Distinct Oral Microbial Communities Based on High-Throughput Sequencing of 16S rRNA Gene Fragments
Source: PLoS Negl Trop Dis. 2014 Dec 4;8(12):e3240. doi: 10.1371/journal.pntd.0003240 (PMC4256271; doi:10.1371/journal.pntd.0003240)
Supplement: Table S4 — Shared OTUs at 97% (on top, in bold) and 99% clustering cut-offs. (DOCX) [file pntd.0003240.s007.docx]

**Table S4:** Shared OTUs at 97% (on top, in bold) and 99% clustering cut-offs

|  | NH | N | C | ANGH | ANG |
| --- | --- | --- | --- | --- | --- |
| NH |  | **309** | **268** | **292** | **297** |
| N | 514 |  | **262** | **321** | **346** |
| C | 471 | 429 |  | **295** | **275** |
| ANGH | 512 | 528 | 528 |  | **358** |
| ANG | 495 | 584 | 474 | 675 |  |
