# Supplementary material for: Noma Affected Children from Niger Have Distinct Oral Microbial Communities Based on High-Throughput Sequencing of 16S rRNA Gene Fragments
Source: PLoS Negl Trop Dis. 2014 Dec 4;8(12):e3240. doi: 10.1371/journal.pntd.0003240 (PMC4256271; doi:10.1371/journal.pntd.0003240)
Supplement: Table S5 — Pairwise sample dissimilarities, calculated using the Bray-Curtis measure, at different levels of OTU identity cut-off. (DOCX) [file pntd.0003240.s008.docx]

**Table S5:** Pairwise sample dissimilarity, with Bray-Curtis distance measure, at different levels of identity cut-off for OTU formation.

| **90 otus** | NH | N | C | ANGH | ANG |
| --- | --- | --- | --- | --- | --- |
| **NH** |  |  |  |  |  |
| **N** | 0.41 |  |  |  |  |
| **C** | 0.35 | 0.59 |  |  |  |
| **ANGH** | 0.23 | 0.36 | 0.33 |  |  |
| **ANG** | 0.41 | 0.23 | 0.51 | 0.33 |  |
|  |  | |  |  |  |
| **97%** | NH | N | C | ANGH | ANG |
| **NH** |  |  |  |  |  |
| **N** | 0.52 |  |  |  |  |
| **C** | 0.47 | 0.68 |  |  |  |
| **ANGH** | 0.35 | 0.47 | 0.43 |  |  |
| **ANG** | 0.56 | 0.36 | 0.63 | 0.44 |  |
|  |  |  |  |  |  |
| **99 otus** | NH | N | C | ANGH | ANG |
| **NH** |  |  |  |  |  |
| **N** | 0.59 |  |  |  |  |
| **C** | 0.55 | 0.74 |  |  |  |
| **ANGH** | 0.47 | 0.58 | 0.55 |  |  |
| **ANG** | 0.63 | 0.47 | 0.69 | 0.52 |  |
