# Supplementary material for: Noma Affected Children from Niger Have Distinct Oral Microbial Communities Based on High-Throughput Sequencing of 16S rRNA Gene Fragments
Source: PLoS Negl Trop Dis. 2014 Dec 4;8(12):e3240. doi: 10.1371/journal.pntd.0003240 (PMC4256271; doi:10.1371/journal.pntd.0003240)
Supplement: Table S6 — Indicator species analysis with comparison of different sample groups. Red indicates enrichment in a disease state (Noma or ANG) and black indicates enrichment in the control or non-disease state. (DOCX) [file pntd.0003240.s009.docx]

**Table S6:** Indicator species analysis with comparison of different sample groups. Red indicates enrichment in a disease state (Noma or ANG) and black indicates enrichment in the control or non-disease state.

| OTU ID | Indicator of | Indicator value | P value | Taxon (Phylum;Class;Order;Family;Genus;Species) |
| --- | --- | --- | --- | --- |
| 629 | ANG | 0.5317 | 0.001 | Bacteroidetes;Bacteroidia;Bacteroidales;Porphyromonadaceae;Porphyromonas;endodontalis |
| 924 | ANG | 0.5283 | 0.004 | Bacteroidetes;Bacteroidia;Bacteroidales;Prevotellaceae;Prevotella;intermedia |
| 744 | ANG | 0.4818 | 0.002 | Spirochaetes;Spirochaetes;Spirochaetales;Spirochaetaceae;Treponema |
| 1063 | ANG | 0.4693 | 0.001 | Firmicutes;Clostridia;Clostridiales;Clostridiaceae |
| 274 | ANG | 0.4679 | 0.001 | Bacteroidetes;Bacteroidia;Bacteroidales;Prevotellaceae;Prevotella;intermedia |
| 1050 | ANG | 0.4369 | 0.008 | Bacteroidetes;Bacteroidia;Bacteroidales;Prevotellaceae;Prevotella |
| 33 | ANG | 0.4002 | 0.029 | Bacteroidetes;Bacteroidia;Bacteroidales;[Paraprevotellaceae];[Prevotella];tannerae |
| 805 | ANG | 0.3958 | 0.009 | Firmicutes;Clostridia;Clostridiales;Peptostreptococcaceae;Filifactor |
| 1166 | ANG | 0.3442 | 0.046 | Bacteroidetes;Bacteroidia;Bacteroidales;Prevotellaceae;Prevotella |
| 833 | ANG | 0.3409 | 0.014 | Firmicutes;Clostridia;Clostridiales;Clostridiaceae |
| 1133 | ANG | 0.3401 | 0.048 | Firmicutes;Clostridia;Clostridiales |
| 717 | ANG | 0.3137 | 0.044 | Proteobacteria;Alphaproteobacteria |
| 563 | ANG | 0.3091 | 0.016 | Spirochaetes;Spirochaetes;Spirochaetales;Spirochaetaceae;Treponema |
| 738 | ANG | 0.3011 | 0.04 | Proteobacteria;Alphaproteobacteria |
| 1201 | ANG | 0.2976 | 0.027 | Bacteroidetes;Bacteroidia;Bacteroidales;Prevotellaceae;Prevotella |
| 955 | ANG | 0.2921 | 0.043 | Bacteroidetes;Bacteroidia;Bacteroidales |
| 574 | ANG | 0.2722 | 0.034 | Bacteroidetes;Bacteroidia;Bacteroidales;Prevotellaceae;Prevotella;intermedia |
| 188 | ANG | 0.25 | 0.036 | Proteobacteria;Alphaproteobacteria |
| 287 | ANG | 0.25 | 0.037 | Bacteroidetes;Bacteroidia;Bacteroidales;Prevotellaceae;Prevotella;intermedia |
| 602 | ANG | 0.25 | 0.017 | Bacteroidetes;Bacteroidia;Bacteroidales;Prevotellaceae;Prevotella |
| 1126 | ANG | 0.25 | 0.039 | Firmicutes;Clostridia;Clostridiales;Veillonellaceae;Selenomonas |
| 873 | ANG | 0.2193 | 0.044 | Proteobacteria |
| 632 | ANGH | 0.4403 | 0.005 | Bacteroidetes;Bacteroidia;Bacteroidales |
| 960 | ANGH | 0.3636 | 0.004 | Proteobacteria;Epsilonproteobacteria;Campylobacterales;Campylobacteraceae;Campylobacter;rectus |
| 44 | ANGH | 0.3589 | 0.026 | Firmicutes;Clostridia;Clostridiales;Lachnospiraceae |
| 1092 | ANGH | 0.3333 | 0.006 | Bacteroidetes;Bacteroidia;Bacteroidales |
| 973 | ANGH | 0.3238 | 0.049 | Bacteroidetes;Flavobacteriia;Flavobacteriales;Flavobacteriaceae;Elizabethkingia |
| 371 | ANGH | 0.307 | 0.013 | Bacteroidetes;Bacteroidia;Bacteroidales;Porphyromonadaceae;Porphyromonas;endodontalis |
| 58 | ANGH | 0.2667 | 0.023 | Bacteroidetes;Flavobacteriia;Flavobacteriales;Flavobacteriaceae;Capnocytophaga;ochracea |
| 324 | ANGH | 0.2292 | 0.048 | Proteobacteria;Gammaproteobacteria;Pasteurellales;Pasteurellaceae;Aggregatibacter;actinomycetemcomitans |
| 1171 | ANGH | 0.2115 | 0.043 | Bacteroidetes;Flavobacteriia;Flavobacteriales;Flavobacteriaceae;Capnocytophaga |
| 460 | Control | 0.783 | 0.001 | Proteobacteria;Gammaproteobacteria;Pasteurellales;Pasteurellaceae;Aggregatibacter |
| 495 | Control | 0.7348 | 0.001 | Bacteroidetes;Flavobacteriia;Flavobacteriales;Flavobacteriaceae;Capnocytophaga |
| 921 | Control | 0.5819 | 0.001 | Bacteroidetes;Flavobacteriia;Flavobacteriales;Flavobacteriaceae;Capnocytophaga;ochracea |
| 1223 | Control | 0.5515 | 0.005 | Proteobacteria;Betaproteobacteria;Neisseriales;Neisseriaceae |
| 677 | Control | 0.5371 | 0.002 | Bacteroidetes;Bacteroidia;Bacteroidales;Porphyromonadaceae;Porphyromonas |
| 596 | Control | 0.5331 | 0.001 | Actinobacteria;Actinobacteria;Actinomycetales;Propionibacteriaceae |
| 272 | Control | 0.5219 | 0.001 | Proteobacteria;Gammaproteobacteria;Cardiobacteriales;Cardiobacteriaceae;Cardiobacterium |
| 1305 | Control | 0.5172 | 0.001 | Bacteroidetes;Flavobacteriia;Flavobacteriales;Flavobacteriaceae;Capnocytophaga |
| 101 | Control | 0.5149 | 0.001 | Proteobacteria;Betaproteobacteria;Neisseriales;Neisseriaceae;Kingella |
| 285 | Control | 0.5118 | 0.001 | Bacteroidetes;Flavobacteriia;Flavobacteriales;Flavobacteriaceae;Capnocytophaga;ochracea |
| 1111 | Control | 0.5052 | 0.004 | Proteobacteria;Betaproteobacteria;Neisseriales;Neisseriaceae;Neisseria |
| 477 | Control | 0.458 | 0.003 | Bacteroidetes;Flavobacteriia;Flavobacteriales;Flavobacteriaceae;Capnocytophaga |
| 497 | Control | 0.4538 | 0.001 | Fusobacteria;Fusobacteria;Fusobacteriales;Leptotrichiaceae;Leptotrichia |
| 293 | Control | 0.4506 | 0.001 | Bacteroidetes;Bacteroidia;Bacteroidales;Porphyromonadaceae;Porphyromonas |
| 1275 | Control | 0.45 | 0.001 | Bacteroidetes;Flavobacteriia;Flavobacteriales;Flavobacteriaceae;Capnocytophaga |
| 1140 | Control | 0.4464 | 0.002 | Proteobacteria;Betaproteobacteria;Burkholderiales;Comamonadaceae;Hylemonella |
| 1022 | Control | 0.4359 | 0.008 | Proteobacteria;Gammaproteobacteria;Pasteurellales;Pasteurellaceae;Haemophilus |
| 273 | Control | 0.4354 | 0.004 | Actinobacteria;Actinobacteria;Actinomycetales;Corynebacteriaceae;Corynebacterium |
| 639 | Control | 0.4213 | 0.001 | Fusobacteria;Fusobacteria;Fusobacteriales;Leptotrichiaceae |
| 723 | Control | 0.4132 | 0.011 | Proteobacteria;Gammaproteobacteria;Pasteurellales;Pasteurellaceae;Aggregatibacter |
| 1276 | Control | 0.3916 | 0.026 | Fusobacteria;Fusobacteria;Fusobacteriales;Leptotrichiaceae;Leptotrichia |
| 150 | Control | 0.3792 | 0.007 | Proteobacteria;Gammaproteobacteria;Pasteurellales;Pasteurellaceae |
| 1260 | Control | 0.3771 | 0.004 | Firmicutes;Bacilli;Gemellales;Gemellaceae |
| 620 | Control | 0.3667 | 0.02 | Bacteroidetes;Flavobacteriia;Flavobacteriales;Flavobacteriaceae |
| 1280 | Control | 0.3662 | 0.012 | Firmicutes;Clostridia;Clostridiales;Lachnospiraceae;Johnsonella |
| 864 | Control | 0.3646 | 0.003 | Proteobacteria;Betaproteobacteria;Neisseriales;Neisseriaceae;Kingella |
| 256 | Control | 0.3601 | 0.006 | Proteobacteria;Betaproteobacteria;Burkholderiales;Comamonadaceae;Hylemonella |
| 1239 | Control | 0.344 | 0.016 | Actinobacteria;Actinobacteria;Actinomycetales;Actinomycetaceae;Parascardovia |
| 1238 | Control | 0.3379 | 0.041 | Proteobacteria;Epsilonproteobacteria;Campylobacterales;Campylobacteraceae;Campylobacter |
| 317 | Control | 0.3333 | 0.005 | Proteobacteria;Gammaproteobacteria |
| 718 | Control | 0.3333 | 0.025 | Actinobacteria;Actinobacteria;Actinomycetales;Micrococcaceae;Rothia;aeria |
| 844 | Control | 0.2903 | 0.018 | Fusobacteria;Fusobacteria;Fusobacteriales;Leptotrichiaceae |
| 1144 | Control | 0.2778 | 0.017 | Proteobacteria;Gammaproteobacteria;Cardiobacteriales;Cardiobacteriaceae;Cardiobacterium;valvarum |
| 1321 | Control | 0.2667 | 0.016 | Bacteroidetes;Bacteroidia;Bacteroidales;Porphyromonadaceae;Porphyromonas |
| 790 | Control | 0.2593 | 0.043 | Bacteroidetes;Bacteroidia;Bacteroidales;Porphyromonadaceae;Porphyromonas |
| 792 | Control | 0.25 | 0.031 | Proteobacteria;Alphaproteobacteria;Rhizobiales;Methylobacteriaceae |
| 938 | Control | 0.25 | 0.045 | Proteobacteria;Alphaproteobacteria;Rhizobiales;Bradyrhizobiaceae;Balneimonas |
| 1286 | Control | 0.2424 | 0.027 | Bacteroidetes;Bacteroidia;Bacteroidales;Prevotellaceae;Prevotella |
| 536 | Control | 0.2381 | 0.041 | Bacteroidetes;Flavobacteriia;Flavobacteriales;Flavobacteriaceae;Capnocytophaga |
| 405 | N | 0.8656 | 0.001 | Firmicutes;Erysipelotrichi;Erysipelotrichales;[Coprobacillaceae];Sharpea |
| 1044 | N | 0.4708 | 0.004 | Bacteroidetes;Bacteroidia;Bacteroidales;Prevotellaceae;Prevotella |
| 667 | N | 0.4656 | 0.004 | Bacteroidetes;Bacteroidia;Bacteroidales |
| 705 | N | 0.4259 | 0.003 | Firmicutes;Clostridia;Clostridiales;Peptostreptococcaceae;Peptostreptococcus |
| 756 | N | 0.4125 | 0.002 | Firmicutes;Clostridia;Clostridiales;Lachnospiraceae;Oribacterium |
| 1064 | N | 0.4074 | 0.007 | Firmicutes;Clostridia;Clostridiales;Veillonellaceae;Dialister;invisus |
| 85 | N | 0.3758 | 0.027 | Firmicutes;Clostridia;Clostridiales;Veillonellaceae |
| 250 | N | 0.363 | 0.01 | Spirochaetes;Spirochaetes;Spirochaetales;Spirochaetaceae;Treponema |
| 770 | N | 0.3367 | 0.019 | Bacteroidetes;Bacteroidia;Bacteroidales;Prevotellaceae;Prevotella;melaninogenica |
| 881 | N | 0.334 | 0.043 | Firmicutes;Clostridia;Clostridiales;Veillonellaceae |
| 438 | N | 0.33 | 0.01 | Bacteroidetes;Bacteroidia;Bacteroidales;Prevotellaceae;Prevotella |
| 820 | N | 0.303 | 0.011 | Spirochaetes;Spirochaetes;Spirochaetales;Spirochaetaceae;Treponema |
| 866 | N | 0.2838 | 0.04 | Firmicutes;Clostridia;Clostridiales;Ruminococcaceae |
| 200 | N | 0.25 | 0.029 | Spirochaetes;Spirochaetes;Spirochaetales;Spirochaetaceae;Treponema |
| 676 | N | 0.25 | 0.028 | Bacteria |
| 907 | N | 0.25 | 0.029 | Bacteroidetes;Bacteroidia;Bacteroidales;[Paraprevotellaceae];[Prevotella] |
| 1124 | N | 0.25 | 0.034 | Tenericutes;Mollicutes;Anaeroplasmatales;Anaeroplasmataceae |
| 1061 | N | 0.2 | 0.043 | Bacteroidetes;Bacteroidia;Bacteroidales |
| 288 | NH | 0.5157 | 0.046 | Firmicutes;Clostridia;Clostridiales;Veillonellaceae;Veillonella;dispar |
| 55 | NH | 0.25 | 0.027 | Proteobacteria;Betaproteobacteria;Burkholderiales;Comamonadaceae;Delftia |
